# Low-Dose Ketamine-Induced Deficits in Arbitrary Visuomotor Mapping in Monkeys

**DOI:** 10.1523/ENEURO.0015-23.2023

**Published:** 2023-06-23

**Authors:** Zhi-Ping Zhao, Chuang Nie, Cheng-Teng Jiang, Sheng-Hao Cao, Kai-Xi Tian, Xin-Yong Han, Shan Yu, Jian-Wen Gu

**Affiliations:** 1 School of Biological Science and Medical Engineering, BeiHang University, Beijing 100191, China; 2 Strategic Support Force Medical Center, Beijing 100101, China; 3 Laboratory of Brain Atlas and Brain-inspired Intelligence, Chinese Academy of Sciences Institute of Automation, Beijing 100190, China; 4 Department of Ophthalmology, First Hospital of Jilin University, Changchun 130021, China; 5 Savaid Medical School, University of Chinese Academy of Sciences, Beijing 100049, China; 6 School of Future Technology, University of Chinese Academy of Sciences, Beijing 100049, China; 7 School of Artificial Intelligence, University of Chinese Academy of Sciences, Beijing 100049, China

**Keywords:** abstract rule, acute, cognition, ketamine, low-dose, visuomotor mapping

## Abstract

Ketamine, an NMDA antagonist, is widely used in clinical settings. Recently, low-dose ketamine has gained attention because of its promising role as a rapid antidepressant. However, the effects of low-dose ketamine on brain function, particularly higher cognitive functions of primate brains, are not fully understood. In this study, we used two macaques as subjects and found that acute low-dose ketamine administration significantly impaired the ability for arbitrary visuomotor mapping (AVM), a form of associative learning (AL) essential for flexible behaviors, including executions of learned stimuli-response contingency or learning of new contingencies. We conducted in-depth analyses and identified intrinsic characteristics of these ketamine-induced functional deficits, including lowered accuracy, prolonged time for planning and movement execution, increased tendency to make errors when visual cues are changed from trial to trial, and stronger impact on combining associative learning and another key higher cognitive function, working memory (WM). Our results shed new light on how associative learning relies on the NMDA-mediated synaptic transmission of the brain and contribute to a better understanding of the potential acute side effects of low-dose ketamine on cognition, which can help facilitate its safe usage in medical practice.

## Significance Statement

This study found that acute low-dose ketamine significantly impaired the ability of arbitrary visual motor mapping, a critical form of associative learning (AL) for flexible behavior, including well-learned stimulus-response contingency or learning of new contingencies. We conducted a thorough analysis and identified intrinsic features of ketamine-induced functional deficits, including decreased accuracy, prolonged planning and motor execution time, increased error tendency when visual cues changed from trial to trial, and stronger impact on combining associative learning and another key higher-order cognitive function, working memory (WM). These findings contribute to a better understanding of the potential acute cognitive side effects of low-dose ketamine and promote its safe use in medical practice.

## Introduction

Ketamine is a well-known NMDA receptor (NMDAR) antagonist, widely used in clinical settings for inducing anesthesia, providing pain relief, and sedation (Pribish et al., 2020). Recently, low-dose ketamine has attracted attention for its promising role as a rapid antidepressant (Shinohara et al., 2021). However, to ensure safe usage, it is crucial to understand how ketamine affects normal brain functions. Accumulating evidence has demonstrated that low-dose ketamine has a detrimental impact on learning and memory (Zhornitsky et al., 2022). Therefore, investigating the effects of ketamine on cognitive functions, such as associative learning (AL) and working memory (WM), is essential to facilitate its appropriate and safe use in medical practice.

Associative learning and working memory are crucial functions that support higher cognition and represent the two pillars of cognitive control. Cognitive control is defined as the ability to acquire and implement the “rules of the game” necessary to achieve a given goal in a specific situation (Miller and Cohen, 2001), which is essential for flexible intended behaviors in primates. Associative learning allows us to associate different sensory stimuli or cues with appropriate behavioral responses (Tót et al., 2022), which is crucial for learning both concrete and abstract “rules of the game” (McGann, 2015). Arbitrary visuomotor mapping (AVM) is a specific form of associative learning that maps visual cues to corresponding motor responses (Murray et al., 2000), such as the green traffic light allowing road crossing while the red one forbids it. The visual feature relevant to AVM is highly flexible, such as shape, color, and number of objects, while the corresponding mapping rule is purely arbitrary, meaning that it can be changed if necessary (Ameli et al., 2011). The flexibility of AVM enables primates to quickly learn and exploit useful contingency rules, facilitating context-dependent adaptive behavior in complex environments (Mansouri et al., 2020). The execution of learned mapping rules and acquisition of new rules through learning in AVM depend heavily on the prefrontal cortex (PFC) and interactions between the PFC and other areas, such as the premotor cortex and hippocampus (Murray et al., 2000; Puig et al., 2014; Gruart et al., 2015; Nakahara et al., 2016; Mansouri et al., 2020; K Namboodiri and Stuber, 2021; Tót et al., 2022). Additionally, NMDARs play a critical role in connecting physiological changes occurring at synapses to the acquisition of AVM (Gruart et al., 2015).

To fully use AVM, it is often necessary to engage another key cognitive function: working memory. Working memory enables the temporary storage and manipulation of information in the brain (Jaffe and Constantinidis, 2021). By combining AVM and working memory (AVM+WM), visual cues (or their meaning) can be stored in the brain, enabling appropriate responses to be chosen even after informative cues have disappeared. This facilitates flexible behavior in complex environments. Similar to AVM, AVM+WM heavily relies on the prefrontal cortex (PFC) and is associated with NMDAR activity (Arnsten and Jin, 2014; Wang and Arnsten, 2015; Lisman, 2017; Lundqvist et al., 2018). Notably, low-dose ketamine can cause working memory impairment (Ma et al., 2018; Sawagashira and Tanaka, 2021; Roussy et al., 2021). However, the impact of ketamine on the ability to combine AVM and WM to guide behavior remains unclear.

Therefore, in this study, we aimed to investigate the effects of low-dose ketamine on the following functional aspects: (1) execution of a learned AVM rule; (2) learning of new AVM rules; and (3) execution of WM-based AVM. To facilitate comparison between different tasks, the same monkeys, ketamine doses, and similar behavioral settings were used. We hope that the results will help delineate the effects of low-dose ketamine on these important higher cognitive functions.

## Materials and Methods

### Animals

The experiment involved two adult male macaques, aged nine and eight years old, with weights of 9 and 10 kg, respectively. All experimental protocols were approved by the Animal Care and Use Committee of the Institute of Automation, Chinese Academy of Sciences. The animals’ physical and psychological well-being was regularly assessed by researchers and veterinarians. Before the experiments, each animal underwent a standard sterile surgical procedure under general anesthesia to implant a headpost (Adams et al., 2007) for restraining the head in a primate chair during the experimental sessions.

### Behavioral tasks

**Figure 1.**
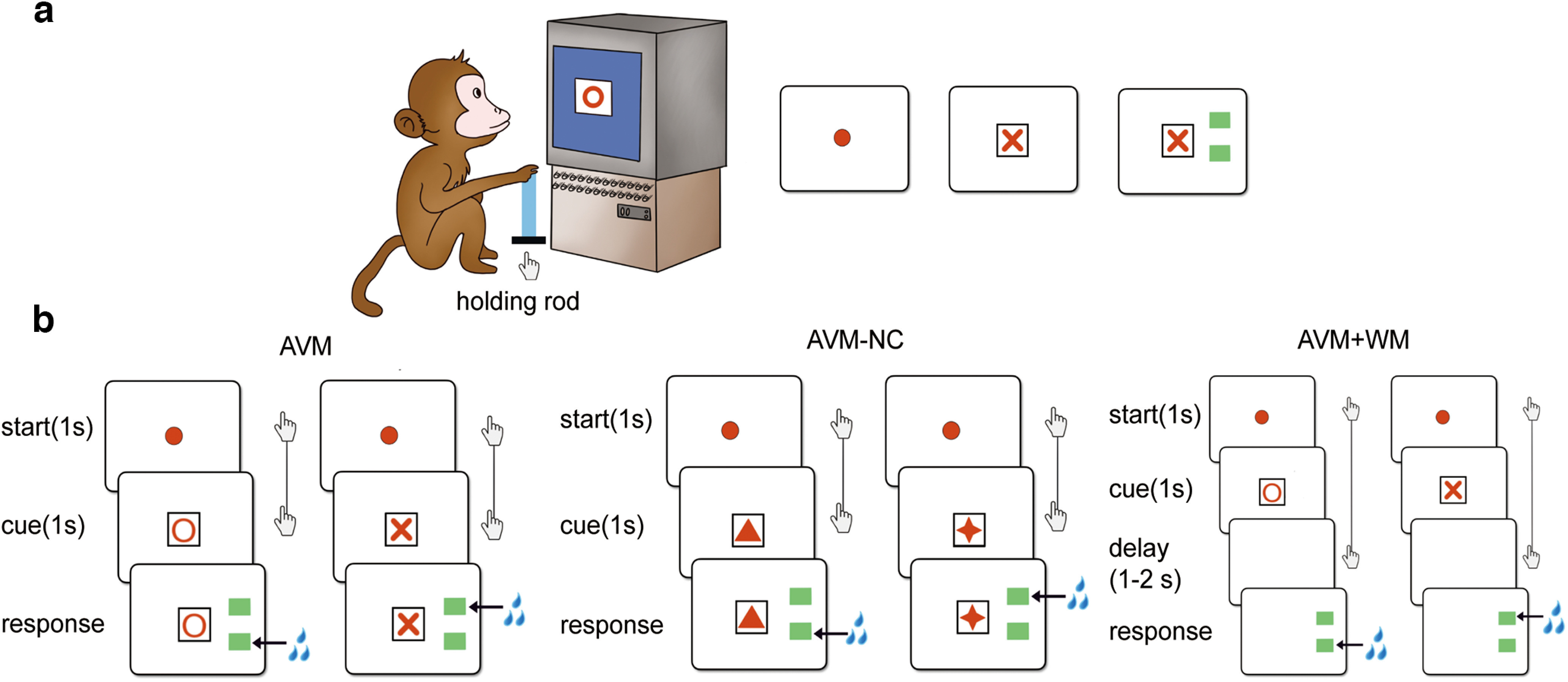
Overall picture of the behavioral tasks. ***a***, Illustration of the experimental setup. ***b***, The three tasks, including AVM, AVM-NC, and AVM+WM, are depicted in diagrams. In the AVM and AVM+WM tasks, visual cues consist of either O or X. In the AVM-NC task, the cues are novel figures that the animals have never seen before. Compared with the AVM and AVM-NC tasks, the AVM+WM task features an additional delay period. The figures with two hands connected by a line indicate that the animals need to maintain their grip on the holding rod. The symbols of triangle and star in AVM-NC represent novel paired cues. The symbol of hand represents the maintain for holding, the lines between represent the holding time. The symbol of water represents the juice reward.

During the experimental sessions, animals were restrained in a primate chair in an isolated room, and they were delivered juice rewards through a tube attached to the chair. The situation was monitored by the experimenter in a nearby chamber. Visual stimuli were presented on a capacitive touch screen (CTS; 17 inches, 1024 × 768-pixel resolution, 60-Hz refresh rate, screen height of 27 cm, and screen width of 34 cm), which was positioned 50 cm from the subjects’ eyes. The touching response of the subjects was registered by the same CTS. In addition, there was a holding rod in front of the primate chair and beside the animals’ right hand (Fig. 1*a*), which was equipped with an infrared sensor to detect the holding response.

There were three tasks during the experiment, including the arbitrary visuomotor mapping (AVM) task, the arbitrary visuomotor mapping with novel cue (AVM-NC) task, and the arbitrary visuomotor mapping with working memory (AVM+WM) task (Fig. 1*b*). All tasks began with a filled red circle appearing at the center of the CTS, instructing the animals to hold the holding rod with their right hands. After the animals held the rod for 1 s, a visual cue consisting of a red letter and a black frame was presented at the center of the CTS for 1 s. For AVM and AVM-NC tasks, after 1 s of cue presentation, two green rectangles (as buttons) appeared on the right side of the CTS, instructing the animals to release the rod and touch one of the buttons according to the visual cue, with their right hands. For the AVM+WM task, after the 1 s of cue presentation, the cue was withdrawn from the screen, followed by a delay period of 1–2 s (Monkey A for 0.8–1.2 s and Monkey Z for 1.8–2.2 s) before the presentation of the buttons as described above, during which a blank screen was presented. In all tasks, correct touching was awarded by 0.5 ml of juice. Incorrect touching, failure to respond within 5 s after the presentation of the buttons, and immature releasing of the holding rod resulted in a punishment of a warning sound (500 ms), withholding the reward, and displaying a black screen for 5 s. The intertrial interval was 2 s.

In both the AVM and AVM+WM tasks, the same visual cues and mapping rules were applied for all trials. Specifically, letter O represented the lower button, and letter X represented the upper button. The animals achieved an accuracy of around 90% through pretraining. In the AVM-NC task, for each daily experimental session, two novel cues that the animals have never seen before were presented, and the corresponding mapping rule needed to be learned on the go. The experimental sessions were completed once the animals achieved 90% accuracy in 50 consecutive trials.

### Testing procedures

In a given daily session, the subjects were randomly assigned to receive either a low dose (0.2 mg/kg) or a high dose (0.8 mg/kg) of ketamine, or saline control, administered intramuscularly in the left arm. Prior research has shown that this dosage of ketamine produces working memory deficits with no anesthetic effects in rhesus monkeys (Roussy et al., 2021). The AVM and AVM+WM tasks consisted of daily sessions that began with a baseline lasting ∼15 min, during which ∼60–70 trials were conducted before the injection. Testing continued for an additional ∼60 min following the injection. In the AVM-NC task, animals received the injection before the start of the session. A minimum 48-h washout period was ensured between consecutive ketamine sessions to avoid cumulative dosing effects.

### Data acquisition and analysis

The experimental procedure was conducted using NIMH Monkeylogic software (Hwang et al., 2019), which also recorded all relevant data for offline analyses. Custom code was written in MATLAB (MathWorks) for all analyses. We recorded behavior performance during 12 saline-AVM sessions (three in Monkey A, nine in Monkey Z), six ketamine (0.2 mg/kg)-AVM sessions (three in Monkey A, three in Monkey Z), 11 ketamine (0.8 mg/kg)-AVM sessions (four in Monkey A, seven in Monkey Z); 26 saline-AVM-NC sessions (14 in Monkey A, 12 in Monkey Z), 13 ketamine (0.2 mg/kg)-AVM-NC sessions (eight in Monkey A, five in Monkey Z), 12 ketamine (0.8 mg/kg)-AVM-NC sessions (six in Monkey A, six in Monkey Z); 23 saline-AVM+WM sessions (nine in Monkey A, 14 in Monkey Z), eight ketamine (0.2 mg/kg)-AVM+WM sessions (four in Monkey A, four in Monkey Z), and eight ketamine (0.8 mg/kg)-AVM+WM sessions (four in Monkey A, four in Monkey Z).

Behavior performance was assessed during three phases: preinjection phase (15 min), early-postinjection phase (0–30 min after injection), and late-postinjection phase (30–60 min after injection) for AVM and AVM+WM tasks. Accuracy was analyzed using the number of complete trials, including both correct and incorrect trials, as the denominator. Incomplete trials, where the monkeys did not respond to the trial start cue, released the holding rod too soon, or did not touch the response buttons, were excluded from the accuracy analysis. The percentage of correctly responded trials was calculated in 5-min bins, resulting in 15 time bins for each daily session. For the AVM-NC task, a moving window consisting of 50 trials was used to calculate the percentage of correctly responded trials for each animal since there was no preinjection phase.

We divided the reaction time (RT) into two components: planning time and movement time. Planning time was defined as the duration between the appearance of response buttons and the release of the holding rod by the animals, while movement time was the period from the release of the holding rod to touching the response buttons. Incomplete trials were excluded from the reaction time analysis.

To investigate the impact of ketamine (0.8 mg/kg) on the relationship between reaction time and accuracy across all tasks, we analyzed all trial data from each session during both the early and late-postinjection phases. We first sorted the trials based on reaction time (i.e., planning time plus movement time) and compared the mean accuracy of the first quarter of trials with the shortest reaction time to the last quarter of trials with the longest reaction time in each task. Additionally, we examined the correlation between reaction time and accuracy for each task separately, based on the analysis of planning and movement time. We divided reaction time into 0.05 s windows and calculated the mean accuracy for each window, then used MATLAB for linear regression analysis to obtain the correlation coefficient.

To assess the impact of ketamine on task engagement in monkeys, we calculated the trial-complete index, which reflects the extent of the animals’ involvement in the task. This index was defined as percentage trial done, which is the ratio of completed trials to all trials (complete and incomplete trials). This metric was calculated separately for each daily session during the early-postinjection phase and compared across three dosages (saline, 0.2 mg/kg ketamine, and 0.8 mg/kg ketamine) separately in each task.

To explore the factors contributing to heightened error rates in the ketamine condition, we classified trials into two categories: those featuring the same visual cue as the preceding correct trial (the “same cue” condition) and those featuring a different visual cue (the “different cue” condition). We then calculated the percentage of incorrect trials in each situation by dividing the number of incorrect trials by the total number of completed trials, during both the early-postinjection and late-postinjection phases, for all tasks in each session. We compared the results across three dosage conditions (saline, 0.2 mg/kg ketamine, and 0.8 mg/kg ketamine). We expanded our analysis to encompass the two and three preceding trials, to eliminate perseveration (a prolonged sequence of trials with subpar performance following a reversal). To facilitate comprehension, we note the same cue and different cue situations as AA and AB, respectively. The first letter denotes the cue for the previous trial, while the second letter represents the cue for the current/analyzed trial. If the previous two/three trials were considered, we can similarly define the same cue situation (AAA/AAAA) and the different cue situations (AAB/AAAB). In all these analyses, we only included the cases in which the previous trials were responded correctly.

In this study, we analyzed data obtained from two monkeys. To ensure clarity and concision in our presentation, we have combined the results from both animals in the main text. However, for readers interested in more detailed information about each individual subject, we have included their respective data in the extended figures.

### Statistical analyses

In order to accurately assess the effect of the drug, we compared the animals’ behavioral performance on the AVM and AVM+WM tasks after ketamine injection to their preinjection baseline within the same recording session. We also compared their performance across three drug conditions (saline, 0.2 mg/kg ketamine, and 0.8 mg/kg ketamine) during the early-postinjection period on the AVM, AVM-NC, and AVM+WM tasks. Statistical significance was assessed using ANOVA and *post hoc* analyses, with Kruskal–Wallis test (K-W) of variance used for non-normally distributed data, and p-values adjusted for multiple comparisons. We used unpaired two-tailed *t* tests to compare the drug effects on planning time, movement time, and mean accuracy between fast and slow trials. For non-normally distributed data, we used the Mann–Whitney test. In addition to MATLAB, we also performed statistical analyses using GraphPad Prism 9.0.0, as appropriate. Results are presented as mean ± SEM unless otherwise specified in all figures.

## Results

### Reduced performance accuracy in all tasks during early-postinjection phase of 0.8 mg/kg ketamine

#### AVM task accuracy

**Figure 2.**
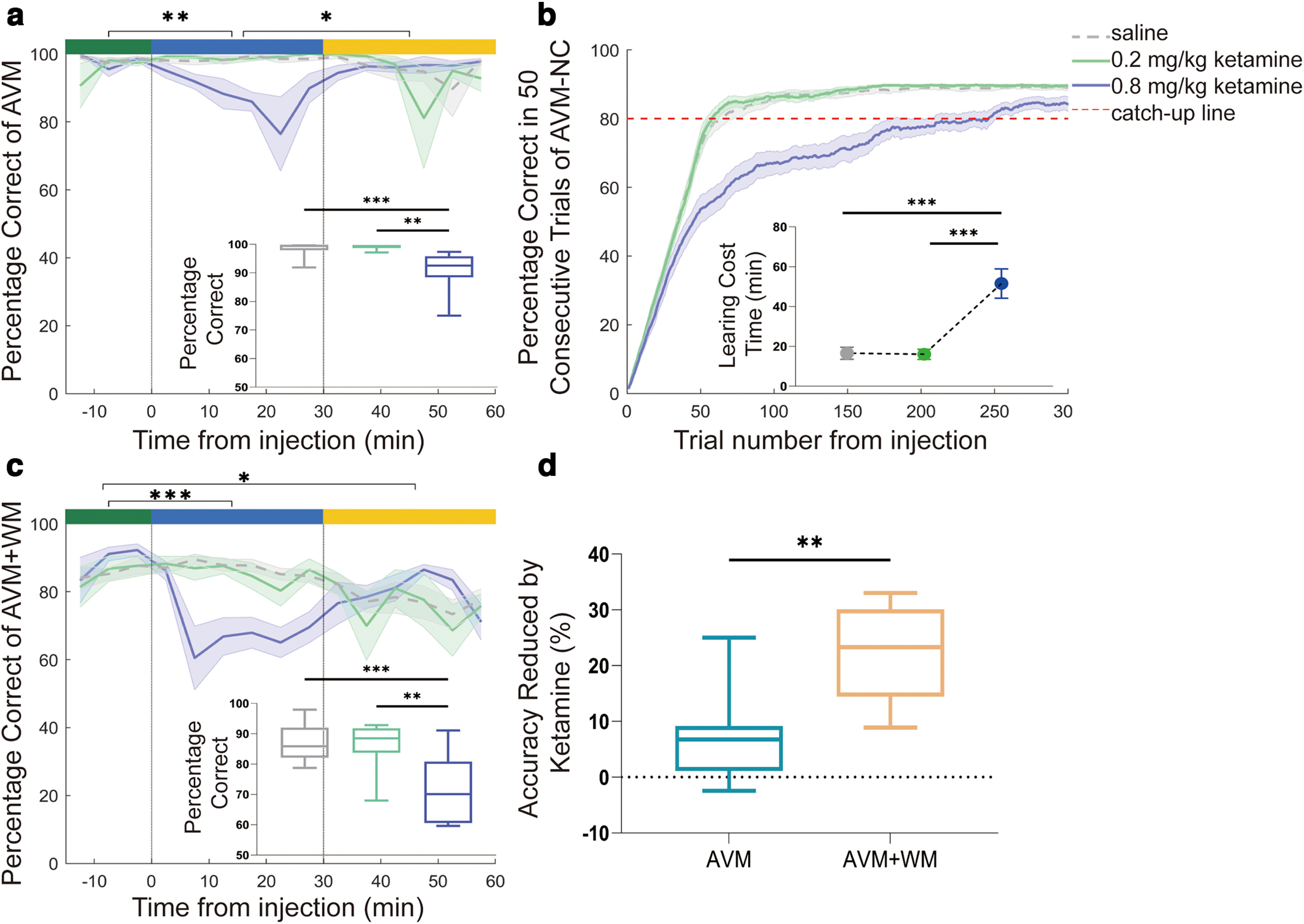
Behavioral performance and comparison of average accuracy in the early-postinjection period for each task. ***a***, Average percent of correct trials in AVM task for saline condition (gray), 0.2 mg/kg ketamine condition (green), and 0.8 mg/kg ketamine condition (blue). The top bars represent data from the preinjection period (green), early-postinjection period (blue), and late-postinjection period (yellow). The box plot in the bottom right corner provides a comparison of the average accuracy during the early-postinjection period for the three conditions such as saline (gray), 0.2 mg/kg ketamine (green), and 0.8 mg/kg ketamine (blue). ***b***, Behavior performance of AVM-NC task. The box plot in the bottom right corner displays the learning cost time, defined as the time it took for the animals to achieve 90% accuracy in 50 consecutive trials, for the three conditions. ***c***, Behavior performance of AVM+WM task. ***d***, This graph compares the effects of 0.8 mg/kg ketamine on the AVM and AVM+WM tasks. The effects are quantified by the percent performance drop, calculated as (A_pre_ − A _early-post_)/A_pre_ %, where A_pre_ and A_early-post_ are the accuracy during the preinjection period and early-postinjection period, respectively. The error bars represent the SEM. Statistical significance is denoted by *p < 0.05, **p < 0.01, and ***p < 0.001. See Extended Data Figure 2-1 for further examination.

To assess the impact of ketamine on the AVM task, we first examined the effects of ketamine on already learned rules. Our findings, shown in Figure 2*a*, revealed that 0.8 mg/kg ketamine significantly decreased AVM accuracy during the early-postinjection period compared with the preinjection period (*p* = 0.0054). However, the impairment was temporary, and the accuracy recovered during the late-postinjection period, becoming significantly higher than the early-postinjection period (*p* = 0.0222) and similar to the preinjection period (*p* = 0.8351). We conducted a cross-condition comparison to account for the effect of long experimental sessions on performance. We found that the saline, 0.2 mg/kg ketamine, and 0.8 mg/kg ketamine conditions significantly differed during the early-postinjection period (K-W, *p* = 0.0003). Specifically, the accuracy of 0.8 mg/kg ketamine was significantly lower than saline (*p* = 0.001) and 0.2 mg/kg ketamine (*p* = 0.0035), while the accuracy of the saline condition was similar to the 0.2 mg/kg ketamine condition (*p* > 0.9999). The individual performance of each subject is presented in Extended Data Figure 2-1*a*. During the late-postinjection period, we found that there were no significant differences between the saline, 0.2 mg/kg ketamine, and 0.8 mg/kg ketamine conditions during the late-postinjection period in the AVM task for both monkeys (Monkey A, K-W, *p* = 0.76; Monkey Z, *p* = 0.98). Therefore, our results demonstrate that 0.8 mg/kg ketamine significantly impairs already learned AVM rules.

10.1523/ENEURO.0015-23.2023.f2-1 Extended Data Figure 2-1 Behavioral performance per subject. ***a***, Percentage of correct trials in the AVM task for saline (gray), ketamine 0.2 mg/kg (green), and ketamine 0.8 mg/kg (blue) sessions. The lower right corner box plot shows the average percentage of correct trials during the early-postinjection period across three conditions. ***b***, Accuracy in 50 consecutive trials in the AVM-NC task for saline, ketamine 0.2 mg/kg, and ketamine 0.8 mg/kg sessions. The lower right corner plot shows the learning cost time across three conditions. ***c***, Percentage of correct trials in the AVM+WM task for saline, ketamine 0.2 mg/kg, and ketamine 0.8 mg/kg sessions. The lower right corner box plot shows the accuracy changed from the preinjection period to early-postinjection period across three conditions. ***d***, Comparison of the effects of ketamine on AVM and AVM+WM tasks for Monkey A and Monkey Z. The effect of 0.8 mg/kg ketamine was quantified as the percent performance drop, calculated by (A_pre_ − A_early-post_)/A_pre_ %, where A_pre_ and A_early-post_ are the accuracy of the preinjection period and early-postinjection period, respectively. The bars represent the declined rate of accuracy. All error bars represent SEM. Statistical significance is denoted by *p < 0.05, **p < 0.01, ***p < 0.001. Download Figure 2-1, TIF file.

#### AVM-NC task accuracy

In this study, we aimed to evaluate the impact of ketamine on abstract rule learning by assessing the ability to learn mapping rules to novel visual cues in the AVM-NC task. As shown in Figure 2*b*, the saline and 0.2 mg/kg ketamine conditions had similar learning performance, with animals gradually reaching 80% accuracy in ∼50 trials (K-W, *p* < 0.0001). However, the 0.8 mg/kg ketamine condition took significantly longer (∼250 trials) to reach 90% accuracy than the saline (*post hoc*, *p* < 0.0001) and 0.2 mg/kg ketamine (*post hoc*, *p* = 0.0009) conditions. The individual performance of each subject is presented in Extended Data Figure 2-1*b*. These findings indicate that 0.8 mg/kg ketamine impairs the ability to learn abstract rules for pairing novel visual cues and rewards.

#### AVM+WM task accuracy

The purpose of this study was to assess the impact of ketamine on AVM+WM task accuracy. Our results, shown in Figure 2*c*, indicated that 0.8 mg/kg ketamine significantly lowered AVM+WM accuracy during the early-postinjection period compared with the preinjection period (*p* = 0.0005). Although the accuracy slightly recovered during the late-postinjection period, it remained significantly lower than the preinjection period (*p* = 0.0463) and similar to the early-postinjection period (*p* = 0.1314). A cross-condition comparison showed that the saline, 0.2 mg/kg ketamine, and 0.8 mg/kg ketamine conditions significantly differed during the early-postinjection period (ANOVA, *F*_(2,36)_ = 13.29, *p* < 0.0001). Specifically, the accuracy of 0.8 mg/kg ketamine was significantly lower than saline (*p* < 0.0001) and 0.2 mg/kg ketamine (*p* = 0.0014), while the accuracy of the saline condition was similar to the 0.2 mg/kg ketamine condition (*p* = 0.9176). The individual performance of each subject is presented in Extended Data Figure 2-1*c*. During the late-postinjection period, we found that there were no significant differences between the saline, 0.2 mg/kg ketamine, and 0.8 mg/kg ketamine conditions during the late-postinjection period in the AVM task for both monkeys (Monkey A, K-W, *p* = 0.85; Monkey Z, *p* = 0.13). These results indicated that the AVM+WM based on the same mapping rule was also significantly impaired by 0.8 mg/kg ketamine.

### Differential effects of ketamine on AVM and AVM+WM tasks performance

We investigated the differential effects of ketamine on the performance of the AVM and AVM+WM tasks by exploring the extent of impairment caused by 0.8 mg/kg ketamine. The effect of ketamine was quantified using the percent performance drop formula (A_pre_ − A_early-post_)/A_pre_ %, where A_pre_ and A_early-post_ are the accuracy of the preinjection phase and early-postinjection phase, respectively. Our findings, presented in Figure 2*d*, demonstrated that the effect of ketamine was significantly higher in the AVM+WM task than in the AVM task (Unpaired *t* test, *t* = 3.935, df = 17, two-tailed, *p* = 0.0011). The individual performance of each subject is presented in Extended Data Figure 2-1*d*. This suggests that ketamine impairs both the application of learned abstract rules and their maintenance in the AVM+WM task.

Our results show that ketamine dose-dependently impairs performance in cognitive tasks, including AVM, AVM+WM, and AVM-NC tasks. Furthermore, the abstract association of pairs was disturbed by ketamine at a dose of 0.8 mg/kg, but not at doses below 0.2 mg/kg. Importantly, our findings suggest that the performance of the AVM+WM task is more vulnerable to the effect of ketamine than the AVM task.

### More errors were induced by ketamine when visual cue changed

**Figure 3.**
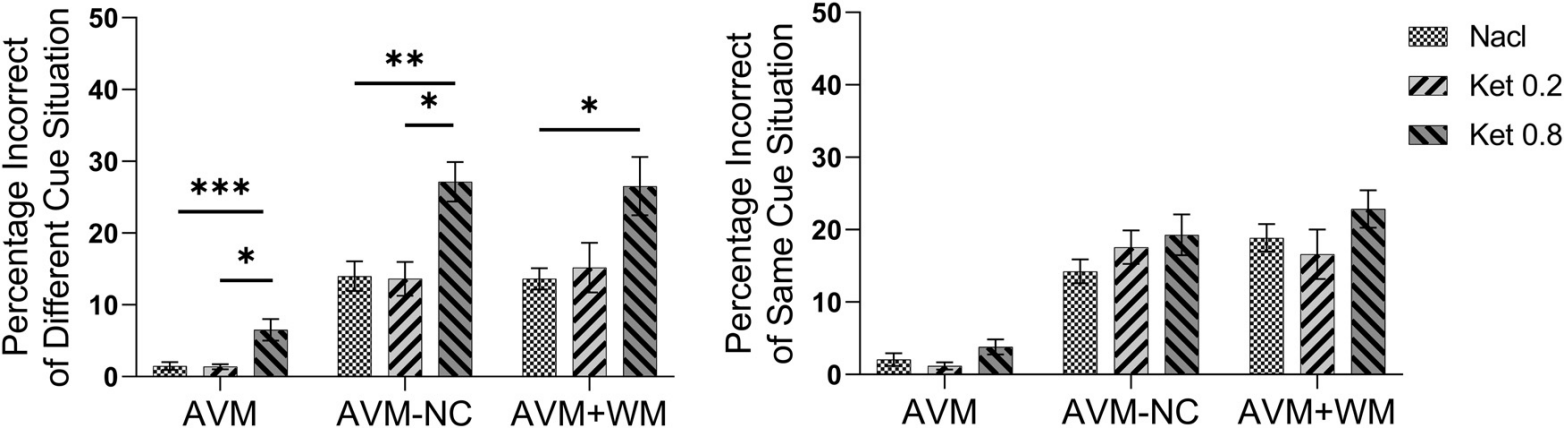
This figure illustrates the percentage of incorrect trials in the same cue and different cue situations during a 60-min period after injection. The average percentage of incorrect trials after injection is presented for the saline group (NaCl), the 0.2 mg/kg ketamine group (Ket 0.2), and the ketamine 0.8 mg/kg group (Ket 0.8). The error bars indicate the SEM. Statistical significance is denoted by *p < 0.05, **p < 0.01, ***p < 0.001. See Extended Data Figures 3-1 and 3-2 for further examination.

To investigate the specificity of ketamine-induced errors, we conducted a study in which we divided the trials into two categories based on whether the visual cues were the same or different from the previous trial. We compared the percentage of incorrect trials in these two situations in each task. As illustrated in Figure 3, we observed that when the cues changed, the administration of 0.8 mg/kg of ketamine resulted in significantly more errors compared with the saline condition in the AVM task (K-W, *p* = 0.0006, *post hoc*, *p* = 0.0009), AVM-NC task (K-W, *p* = 0.0017, *post hoc*, *p* = 0.0020), and AVM+WM task (K-W, *p* = 0.0273, *post hoc*, *p* = 0.0252). Additionally, we observed that 0.8 mg/kg ketamine induced significantly more error trials than the 0.2 mg/kg ketamine condition in the AVM task (K-W, *p* = 0.0008, *post hoc*, *p* = 0.0222) and AVM-NC task (K-W, *p* = 0.0017, *post hoc*, *p* = 0.0104). No significant differences were observed between the saline and 0.2 mg/kg ketamine conditions in each task. In the same cue situation, as shown in Figure 3, there was no significant difference among saline, 0.2 mg/kg, and 0.8 mg/kg ketamine conditions in all tasks. The individual performance of each subject is presented in Extended Data Figure 3-1. These findings suggested that 0.8 mg/kg ketamine induced increased errors when visual cues changed from the immediate past.

10.1523/ENEURO.0015-23.2023.f3-1 Extended Data Figure 3-1 The bad performance in two situations for per subject. The bad performance of trials in different situations per subject. By dividing trials into different cue situations and same cue situation adjusted by the cues was changed or unchanged between two adjacent trials during 60 min after injection. ***a***, Average wrong percentage of trials in the different cue situation after injection for saline (gray), 0.2 mg/kg ketamine (green), and 0.8 mg/kg ketamine trials (blue). ***b***, Average wrong percentage of trials in the same cue situation after injection for saline (gray), 0.2 mg/kg ketamine (green), and 0.8 mg/kg ketamine trials (blue). All error bars are SEM. *p < 0.05, **p < 0.01, ***p < 0.001. Download Figure 3-1, TIF file.

10.1523/ENEURO.0015-23.2023.f3-2 Extended Data Figure 3-2 The figure depicts the percentage of incorrect trials across three trial-forms (AB, AAB, and AAAB) for each task under the 0.8 mg/kg ketamine condition. In the AAB and AAAB trial-forms, the cue from the previous trials that had been responded correctly was denoted as A, while the cue from the analyzed trial was denoted as B. The error bars correspond to the SEM. Download Figure 3-2, TIF file.

To account for the impact of perseveration on the study outcomes, we analyzed the percentage of incorrect trials across three trial-forms (AB, AAB, and AAAB) of each task, while administering 0.8 mg/kg ketamine. Our findings suggest that there were no significant differences between the three task forms when ketamine was given at 0.8 mg/kg (see Extended Data Fig. 3-2). These findings suggest that the observed increase in errors was attributable to trial switch effects rather than perseveration. Notably, if perseveration had been the underlying factor, the percentage of incorrect trials in the AAB or AAAB form would have been higher than that in the AB form.

### Differential effects of ketamine on planning and movement during the response stage of the early-postinjection period

**Figure 4.**
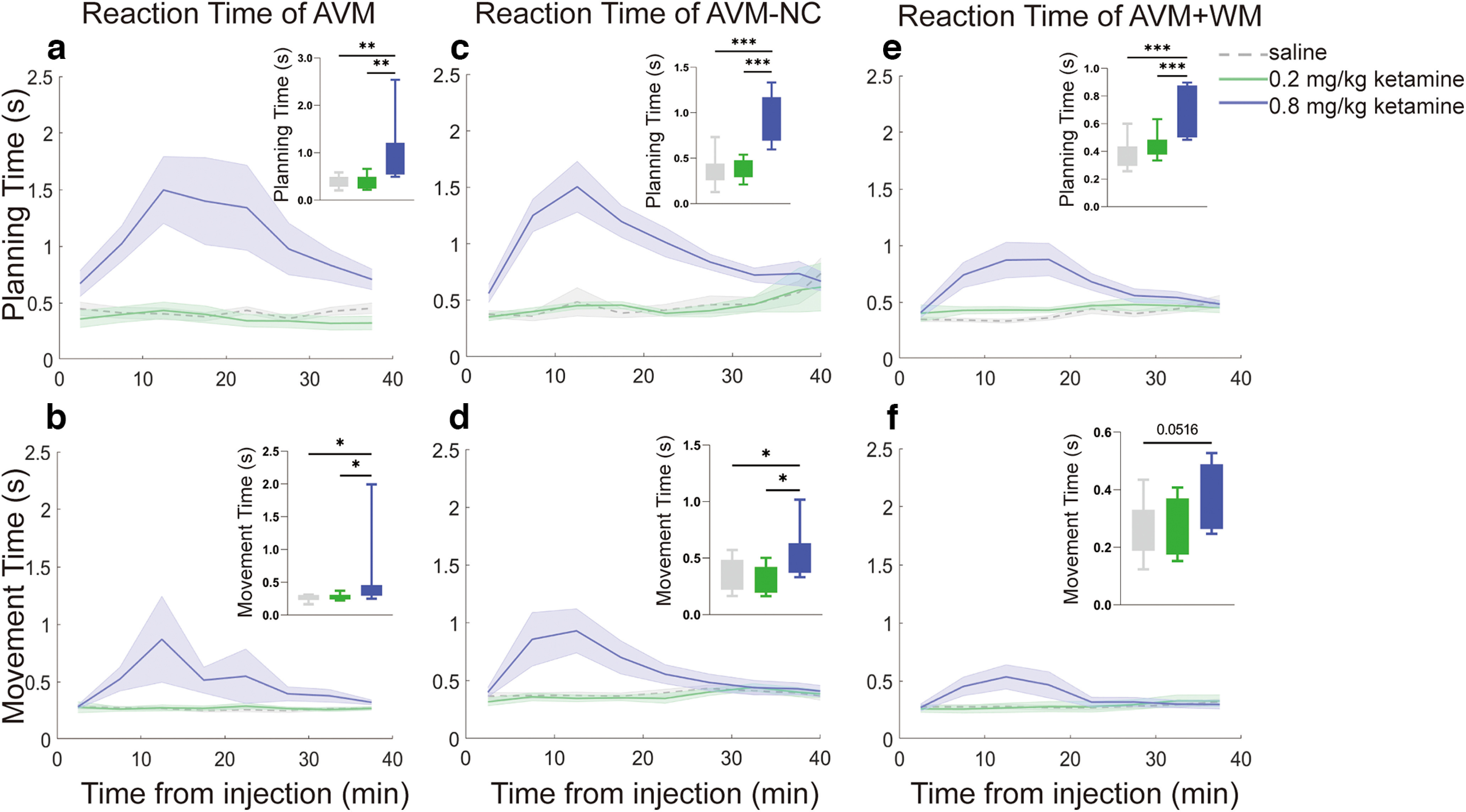
The planning time and movement time in the early-postinjection period for each task. ***a***, Planning time of the response stage of the AVM task for different groups: saline (gray), 0.2 mg/kg ketamine (green), and ketamine 0.8 mg/kg (blue). The box plot in the top right corner shows the average time (s) for each group. ***b***, Movement time of the AVM task. ***c***, Planning time of the AVM-NC task. ***d***, Movement time of the AVM-NC task. ***e***, Movement time of the AVM+WM task. ***f***, Movement time of the AVM+WM task. All error bars represent the SEM. Statistical significance is denoted by *p < 0.05, **p < 0.01, ***p < 0.001. See Extended Data Table 4-1 and Extended Data Figure 4-1 for further examination.

To elucidate the impact of ketamine on the planning and execution of movement, we divided the reaction time into two distinct components: planning time and movement time. As shown in Figure 4, during the early-postinjection stage, both planning and movement times were significantly prolonged to over 1 s under the influence of 0.8 mg/kg ketamine, except for the AVM+WM task. Conversely, planning and movement times remained below 0.5 s in the saline and 0.2 mg/kg ketamine conditions. Our results indicated that both the planning and movement times were substantially lengthened by 0.8 mg/kg ketamine in comparison to both the saline and 0.2 mg/kg ketamine conditions, and this finding was statistically significant in each task. Conversely, no significant difference was observed between the saline and 0.2 mg/kg ketamine conditions (see Extended Data Table 4-1 for statistical details). The individual performance of each subject is presented in Extended Data Figure 4-1.

10.1523/ENEURO.0015-23.2023.f4-1 Extended Data Figure 4-1 Planning and movement time in early-postinjection per subject. Planning time and movement time performance of early-postinjection period in AVM, AVM-NC and AVM+WM task over saline (gray), 0.2 mg/kg ketamine (green) and 0.8 mg/kg ketamine (blue) conditions for Monkey A and Monkey Z. The higher right corner box plot was the average planning time or movement time in the early-postinjection period over saline (gray), 0.2 mg/kg ketamine (green) and 0.8 mg/kg ketamine (blue) conditions. All error bars are SEM. *p < 0.05, **p < 0.001, ***p < 0.001. Download Figure 4-1, TIF file.

10.1523/ENEURO.0015-23.2023.t4-1 Extended Data Table 4-1 Statistics for the Figure 4 in main text. Download Table 4-1, PDF file.

**Figure 5.**
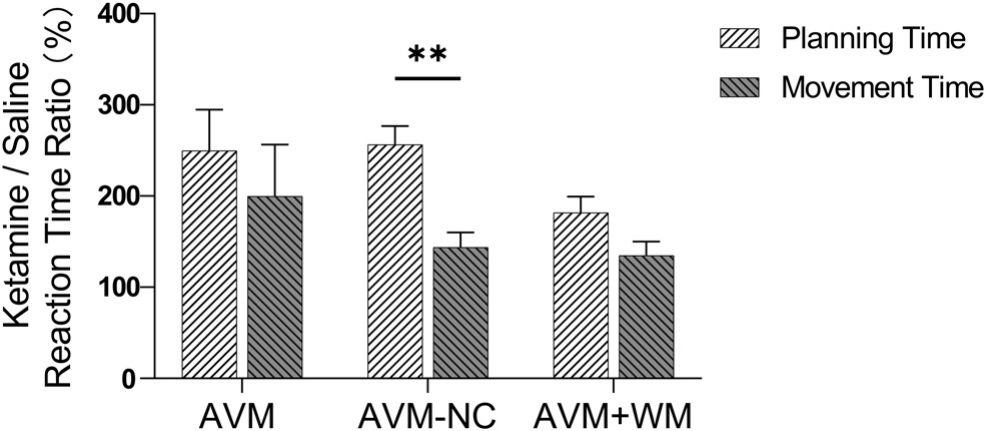
Comparison of planning time and movement time during the early-postinjection period of each task. To assess the effects of 0.8 mg/kg ketamine on planning time and movement time, we calculated the prolonged index for each task. The prolonged index is defined as the ratio of T_plan-0.8_/T_plan-saline_ and T_move-0.8_/T_move-saline_, where T_plan-0.8_ (T_move-0.8_) and T_plan-saline_ (T_move-0.8_) denotes the mean planning (movement) time under the 0.8 mg/kg ketamine and saline conditions, respectively. All error bars are SEM. Statistical significance is denoted by *p < 0.05, **p < 0.01. See Extended Data Figure 5-1 for further examination.

To compare the extent to which 0.8 mg/kg ketamine impairs planning time and movement time, we calculated the prolonged index in each task, i.e., the T_plan-0.8_/T_plan-saline_ and T_move-0.8_/T_move-saline_, where T_plan-0.8_ (T_move-0.8_) and T_plan-saline_ (T_move-0.8_) denotes the mean planning (movement) time under the 0.8 mg/kg ketamine and saline conditions, respectively. Our analysis revealed a general trend that planning time was more affected than movement time, although this difference only reached statistical significance in the AVM-NC task (paired *t* test, *t* = 4.299, df = 12, two-tailed, *p* = 0.0017; Fig. 5). The individual performance of each subject is presented in Extended Data Figure 5-1. These findings suggest that ketamine prolongs reaction times by interfering with both planning and movement, with planning being more severely affected, particularly in the AVM-NC task which involves learning novel visual-motor mapping rules.

10.1523/ENEURO.0015-23.2023.f5-1 Extended Data Figure 5-1 Comparison of planning time and movement time during the early-postinjection period of each task per subject. To assess the effects of 0.8 mg/kg ketamine on planning time and movement time, we calculated the prolonged index for all each task. The prolonged index is defined as the ratio of T_plan-0.8_/T_plan-saline_ and T_move-0.8_/T_move-saline_, where T_plan-0.8_ (T_move-0.8_) and T_plan-saline_ (T_move-0.8_) denotes the mean planning (movement) time under the 0.8 mg/kg ketamine and saline conditions, respectively. All error bars are SEM. Statistical significance is denoted by *p < 0.05, **p < 0.01. Download Figure 5-1, TIF file.

**Figure 6.**
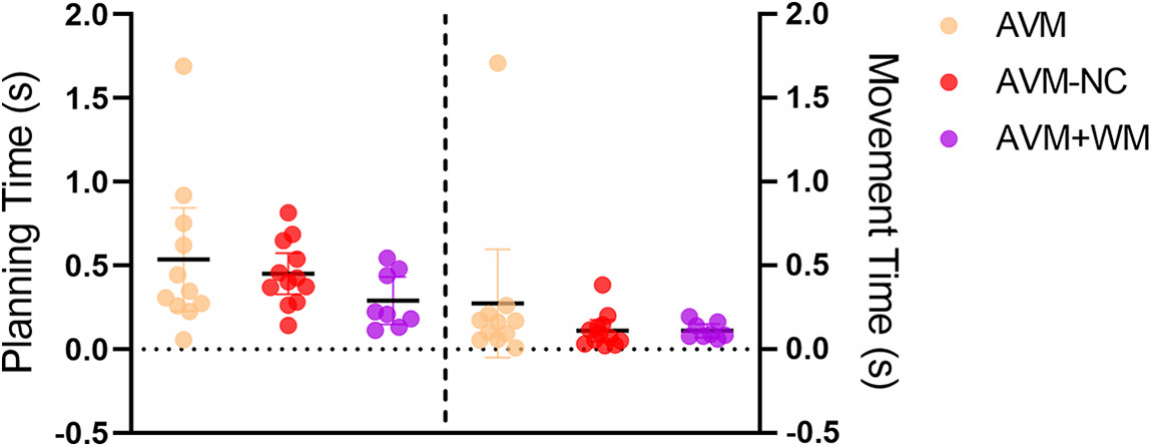
The prolonged change of planning time and movement time under 0.8 mg/kg ketamine conditions relative to saline conditions. The dots in the figure indicate the change in planning (movement) time relative to saline at a dose of 0.8 mg/kg during the early-postinjection phase of the task. See Extended Data Figures 6-1 and 6-2 for further examination. AVM, arbitrary visuomotor mapping task; AVM-NC, arbitrary visuomotor mapping the with novel cue task; AVM+WM, arbitrary visuomotor mapping with working memory task.

To examine the significance of the effect in prolonging planning and movement times among the AVM, AVM-NC and AVM+WM tasks, we analyzed the prolonged part of planning time and movement time with 0.8 mg/kg ketamine and performed statistical analysis across the three tasks. Specifically, we calculated the average planning time and movement time of each session in the early-postinjection and obtained the prolonged change relative to saline conditions by subtracting the values with 0.8 mg/kg ketamine by the mean values of all saline conditions. Our results showed that there was no significant difference across the three tasks in planning time (ANOVA, *F*_(2,28)_ = 1.450, *p* = 0.2516) and movement time (K-W, *p*= 0.3506), as illustrated in Figure 6. The individual performance of each subject is presented in Extended Data Figure 6-1. However, under the 0.8 mg/kg ketamine condition, the prolonged change in planning and movement time for the AVM+WM task indeed showed a downward trend compared with the AVM and AVM-NC tasks.

10.1523/ENEURO.0015-23.2023.f6-1 Extended Data Figure 6-1 The prolonged change of planning time and movement time under 0.8 mg/kg ketamine conditions relative to saline conditions. The dots in the figure indicate the change in planning (movement) time relative to saline at a dose of 0.8 mg/kg during the early-postinjection phase of the task. Download Figure 6-1, TIF file.

10.1523/ENEURO.0015-23.2023.f6-2 Extended Data Figure 6-2 The planning time of early and late saline sessions in the AVM-NC task. The mean interval time between early and late sessions was 14.3 d. ***a***, The mean planning time of both monkeys between early (blue) and late (orange) sessions. ***b***, ***c***, The performance of Monkey A and Monkey Z. Download Figure 6-2, TIF file.

Additionally, to investigate the chronic effect of low-dose ketamine as used in our experiment, we analyzed the performance of three early and three late sessions under saline condition in the AVM-NC task (the mean interval time was 14.3 d). We took the mean values of each session, and used estimation statistics (Ho et al., 2019) to examine the differences between the three early and three late sessions. Our results indicated that planning time in the late sessions was significantly higher than in the early sessions (see Extended Data Fig. 6-2). The mean difference in planning time between the early and late sessions for both subjects was 0.186 s (95% CI = 0.066–0.335 s, *p* = 0.033). Similar trends were observed in Monkey A (mean difference was 0.199 s, 95% CI = 0.042–0.375 s, *p* = 0.092) and Monkey Z (mean difference was 0.173 s, 95% CI = 0.108–0.257 s, *p* = 0.065). However, we observed no significant changes in accuracy, movement time, and percentage trial done after approximately two weeks of intermittent ketamine administration (with at least a 48-h interval between them). In addition, we observed no significant differences in the accuracy, planning time, movement time, and trial done percentage in the AVM and AVM+WM tasks. Thus, in our results, ketamine exhibited no detectable chronical effects except for the planning time in the AVM-NC task.

### Reaction time and accuracy were negatively correlated under ketamine

**Figure 7.**
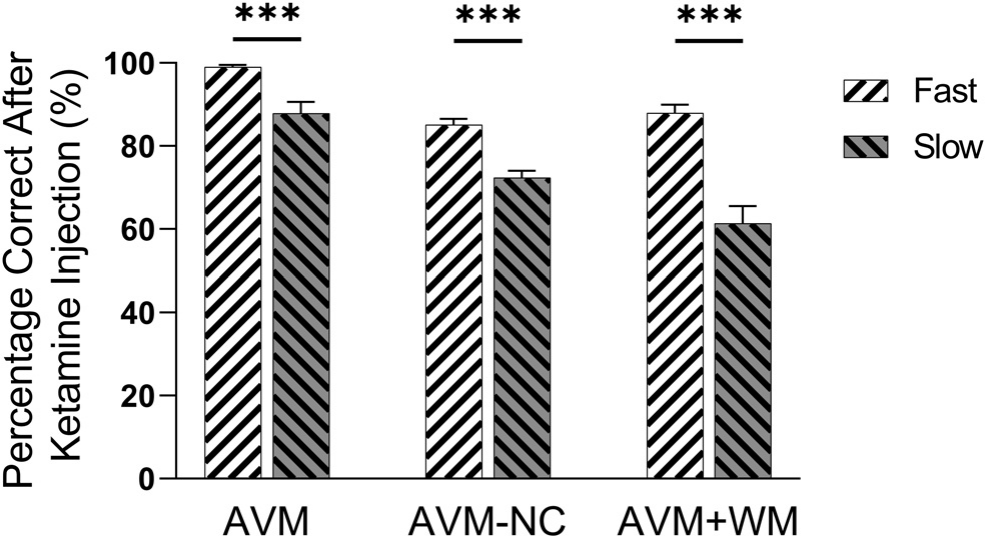
The accuracy comparison of the fast response trials and slow response trials. Trials were rearranged based on reaction time (planning time plus the movement time) following an injection of 0.8 mg/kg ketamine during a 60-min period. Specifically, trials were divided into two groups within each daily session: the fast response group (comprising the first quart of trials with the shortest reaction time) and the slow response group (comprising the last quart of trials with the longest reaction time). The figure shows a comparison of the accuracy between the fast response group (Fast) and the slow response group (Slow). All error bars correspond to the SEM. Significance levels are denoted as follows: *p < 0.05, **p < 0.01, and ***p < 0.001. See Extended Data Figure 7-1 for further examination.

To investigate the potential relationship between impaired accuracy and prolonged reaction time under the 0.8 mg/kg ketamine condition, we compared the accuracy of fast and slow trials separately in each task. Specifically, we compared the accuracy of the quarter of trials with the shortest total reaction time (i.e., fast trials) to that of the quarter of trials with the longest total reaction time (i.e., slow trials). Our results indicated a significant difference in accuracy between fast and slow reaction time trials in in each task: AVM (Mann–Whitney test, two-tailed, *p* < 0.0001), AVM+WM (unpaired *t* test, *t* = 5.816, df = 14, two-tailed, *p* < 0.0001), and AVM-NC (unpaired *t* test, *t* = 5.8, df = 22, two-tailed, *p* < 0.0001; see Fig. 7). The individual performance of each subject is presented in Extended Data Figure 7-1.

10.1523/ENEURO.0015-23.2023.f7-1 Extended Data Figure 7-1 The accuracy comparison of the fast response trials and slow response trials per subject. Trials were rearranged based on reaction time (planning time plus the movement time) following an injection of 0.8 mg/kg ketamine during a 60-min period. Specifically, trials were divided into two groups within each daily session: the fast response group (comprising the first quart of trials with the shortest reaction time) and the slow response group (comprising the last quart of trials with the longest reaction time). The figure shows a comparison of the accuracy between the fast response group (Fast) and the slow response group (Slow). All error bars correspond to the SEM. Significance levels are denoted as follows: *p < 0.05, **p < 0.01, and ***p < 0.001. Download Figure 7-1, TIF file.

**Figure 8.**
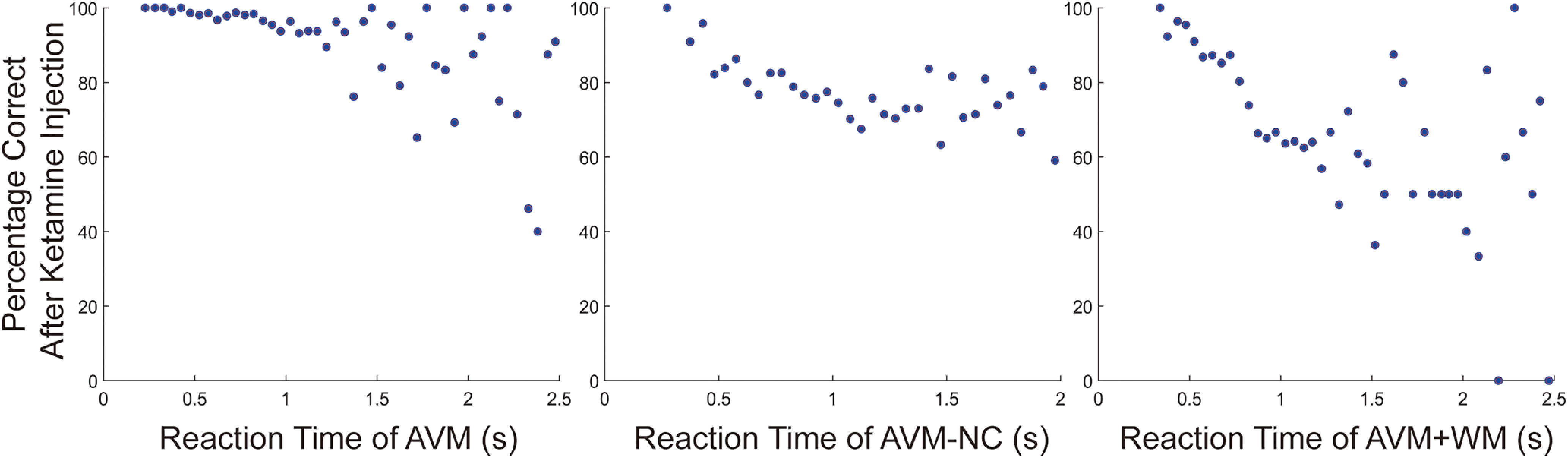
The correlation between the accuracy and reaction time. The dots in the figure represent the corresponding average accuracy within the reaction time. See Extended Data Figure 8-1 for further examination.

Subsequently, we conducted further analysis to investigate the correlation between accuracy and reaction time of trials after 0.8 mg/kg ketamine administration. Based on previous results (as shown in Fig. 4), we selected trials with reaction time <2.5 s for AVM and AVM+WM tasks and those with reaction time <2.0 s for AVM-NC task. Our results indicate a negative correlation between reaction time and accuracy after administration of 0.8 mg/kg ketamine for all tasks (see Fig. 8). Specifically, the correlation coefficient was −0.4576 for AVM task (*p* < 0.001), −0.6016 for AVM-NC task (*p* < 0.001), and −0.4257 for AVM+WM task (*p* < 0.001). Taken together, these findings suggest a negative correlation between accuracy and reaction time in the tasks we investigated following the administration of 0.8 mg/kg ketamine. The individual performance of each subject is presented in Extended Data Figure 8-1.

10.1523/ENEURO.0015-23.2023.f8-1 Extended Data Figure 8-1 The correlation between the accuracy and reaction time after administrating 0.8 mg/kg ketamine per subject. The blue dots in the figure represent the corresponding average accuracy within the reaction time. Download Figure 8-1, TIF file.

### Effects of ketamine on task engagement during the early-postinjection period

**Figure 9.**
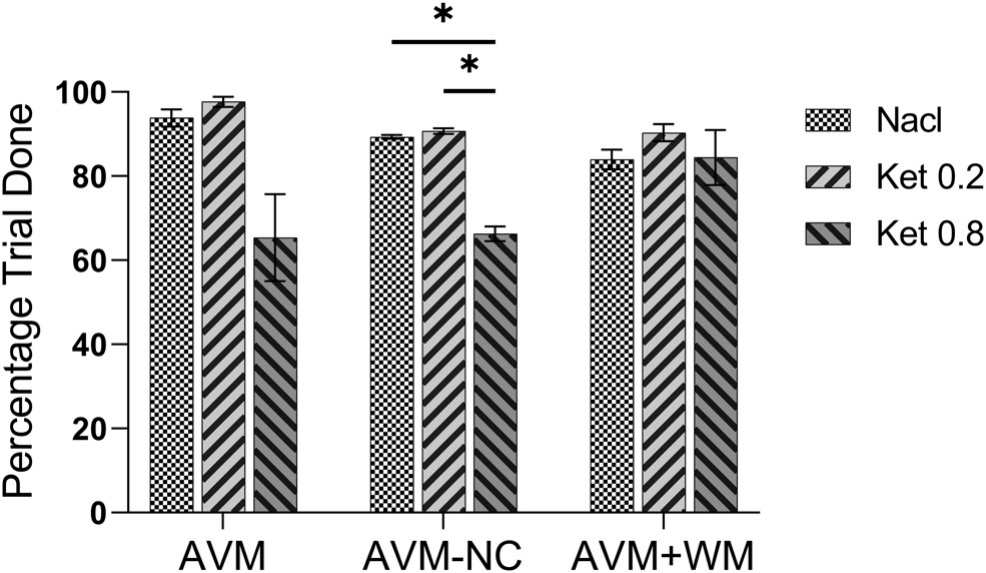
The percentage of complete trials after injection. The percentage trial done was defined as the ratio of completed trials to all trials (complete and incomplete trials). This figure shows the percentage of complete trials in early-postinjection phase under saline (Nacl), 0.2 mg/kg ketamine (Ket 0.2), and 0.8 mg/kg ketamine (Ket 0.8) in each task. The error bars indicate the SEM. Statistical significance is denoted by *p < 0.05. See Extended Data Figure 9-1 for further examination.

Our results, as shown in Figure 8, indicate that there was no significant difference in the trial-complete index between saline, 0.2 mg/kg ketamine, and 0.8 mg/kg ketamine in the AVM task (K-W, *p* = 0.1291) and AVM+WM task (one-way ANOVA, *F*_(2,36)_ = 2.13, *p* = 0.1336). However, the trial-complete index was significantly reduced with 0.8 mg/kg ketamine in the AVM-NC task compared with saline (K-W, *p* = 0.0139; *post hoc*, *p* = 0.0234) and 0.2 mg/kg ketamine (*post hoc*, *p* = 0.0315; as shown in Fig. 9). The individual performance of each subject is presented in Extended Data Figure 9-1. These findings suggest ketamine reduced task engagement particularly in the AVM-NC task which involves learning novel visual-motor mapping rules.

10.1523/ENEURO.0015-23.2023.f9-1 Extended Data Figure 9-1 The percentage of complete trials after injection per subject. The percentage trial done was defined as the ratio of completed trials to all trials (complete and incomplete trials). This figure shows the percentage of complete trials in early-postinjection phase under saline (Nacl), 0.2 mg/kg ketamine (Ket 0.2), and 0.8 mg/kg ketamine (Ket 0.8) in each task. The error bars indicate the SEM. Statistical significance is denoted by *p < 0.05. Download Figure 9-1, TIF file.

## Discussion

In this study, we conducted an in-depth analysis of the effects of low-dose ketamine, specifically subanesthetic doses, on AVM in monkeys. Our results demonstrate profound ketamine-induced behavioral deficits, including: (1) decreased accuracy in three tasks, including the AVM, AVM+WM, and AVM-NC tasks, with a stronger impact in the AVM+WM task than the AVM task; (2) prolonged reaction times, particularly in planning time rather than movement time; (3) increased reaction time during daily sessions in trials with lower accuracy; (4) induced more errors when the visual cue changes; and (5) reduced the ratio of completed trials to all trials in AVM-NC task.

Our study shows that ketamine impaired animals’ performance in AVM, AVM+WM, and AVM-NC tasks at the dose of 0.8 mg/kg but not 0.2 mg/kg, with the latter condition exhibiting similar performance compared with the saline condition. The does dependency is consistent with previous findings that ketamine induces acute impairment of spatial WM tasks above 0.25 mg/kg (Roussy et al., 2021).

In our experiments, the AVM task was trained on the animals with the same visual cues for over a half year before this experiment. It indicates that ketamine impairs the retrieval of well-learned abstract rules in the AVM task, which is in line with a previous study showing that anesthetic ketamine impaired the retrieval of information from post-training memory components (Boultadakis and Pitsikas, 2011). The ketamine-induced impairments in WM have been reported in many previous studies in rodents (Moosavi et al., 2012; Mathews et al., 2018; Pitsikas and Carli, 2020), monkeys (Condy et al., 2005; Skoblenick and Everling, 2012; Blackman et al., 2013; Roussy et al., 2021; Sawagashira and Tanaka, 2021) and humans (Ahn et al., 2003; Honey et al., 2004; Koychev et al., 2017). In this study, we found that the low doses of ketamine consistently reduced the correct percentage over 1 h in both AVM and AVM+WM tasks. At a point of ∼15 min after ketamine injection, the performance declined to almost the chance level (50%) then it started to recover. In addition, we found that ketamine induced worse performance in the AVM+WM task compared with the AVM task. It indicates that the effects of ketamine in WM can be further combined with its impact on AVM, resulting in a more severe impairment in the AVM+WM task. Importantly, our results in the AVM-NC task show that ketamine not only affects retrieving the well-learned visuomotor mapping rules, but it also impairs the ability to acquire new ones. One may argue that a limitation of our study was that we may have not been able to observe animals’ gaze on the visual stimuli and monkeys may be not able to see the monitor properly. However, we recorded electrophysiological signals by chronically implanted microelectrode arrays in the PFC, dorsal premotor cortex (PMd) and the hippocampus in both animals, which we analyzed to address this concern. Specifically, we analyzed the event-related potentials (ERPs) in the PFC and PMd using electrophysiological signals recorded in one channel during the cue phase of the AVM task under the 0.8 mg/kg ketamine condition. We specifically compared the ERPs of all incorrect trials among the preinjection, early-injection, and late-postinjection phases. We found that in both the PFC and the PMd, EPRs calculated based on data collected among these three phases were highly similar, indicating similar early visual processing. It suggested that 0.8 mg/kg ketamine did not affect the monkeys’ ability to attend and to see the cue. Overall, these results suggest that ketamine has a profound impact on key higher cognitive functions, including retrieving and executing learned rules, as well as learning new rules in AVM, and the ability to combine AVM and WM to support more flexible behavioral responses. It is worth noting that the ERPs were not directly measured from visual areas. Consequently, the lack of eye tracking and visual ERP data restricts our ability to fully explore the animals’ perceptual processes in response to visual stimuli.

One possible explanation for the negative impact on learning/performance is through NMDA receptor blockade. However, ketamine may also exert its effects through other mechanisms. For example, ketamine can activate the GABAergic system (Zhang et al., 2021) and increase dopamine release (Kokkinou et al., 2018). As for WM, studies have done and shown that a metabolite of ketamine led to activation of rapamycin (mTOR) signaling (Yang et al., 2018). In addition, ketamine can increase the level of activity in neurons (Wang et al., 2013; Ma et al., 2018; Jang and MacIver, 2021; Roussy et al., 2021), leading to increased noise and impaired signal (Ma et al., 2015). The prefrontal cortex (PFC) has been reported to have a strong relationship with the formation and usage of abstract rules (Seamans et al., 2008; Otani et al., 2015; Vijayraghavan et al., 2017; Mansouri et al., 2020), and it is also well known that the PFC is a key area for WM (Miller and Cohen, 2001). Given the widespread existence of NMDA receptors in the PFC, it would be informative for future studies to investigate whether and how interference with the NMDA system in the PFC can mediate the behavioral effects observed in our study. Currently, little is known in the literature about how ketamine affects the learning process itself in the context of associated learning tasks. Therefore, our results provide new evidence indicating that NMDA may play a key role in learning abstract rules with novel visual cues.

In contrast to previous studies that used eye gaze tasks (Wang et al., 2013; Ma et al., 2018; Sawagashira and Tanaka, 2021), we used more naturalistic tasks to investigate the effects of ketamine on motor planning and execution, by separately analyzing planning and movement time. Our findings demonstrate that ketamine impairs both planning and movement time in AVM, AVM+WM, and AVM-NC tasks. Interestingly, in the AVM-NC task, ketamine had a greater impact on planning time, which may involve higher cognitive processes such as decision-making, compared with movement time, which mainly involves the execution of a planned motor action at the response epoch. Similarly, the AVM and AVM+WM tasks also exhibited a similar trend. While previous studies have reported the effect of ketamine in prolonging reaction time, such as Ma et al. (2018) reported a significant increase in saccadic reaction time in rhesus macaque monkeys during an AVM task; Sawagashira and Tanaka (2021) found reduced saccadic velocity under the influence of ketamine; and Roussy et al. (2021) reported increased reaction time in a visuospatial WM task. However, none of these studies have separately analyzed planning and movement time, particularly in naturalistic tasks. Our results suggest that ketamine may have a more pronounced effect on delaying movement planning and decision-making processes, as opposed to the process of movement execution, particularly in the AVM-NC task, which involves learning novel visual-motor mapping rules. One possible explanation for this is that ketamine effects on learning novel visual-motor mapping rules rather than familiar mapping rules. It is well known that the premotor cortex coordinates with the PFC in conducting rule-based responses (Vallentin et al., 2012), implying that the ketamine may have effects on PFC and premotor cortex area.

Under the condition of administering 0.8 mg/kg of ketamine, there was a decrease in the prolonged change in planning and movement time for the AVM+WM task compared with the AVM and AVM-NC tasks (Fig. 6). One possible explanation for this phenomenon is that task difficulty is a contributing factor. Specifically, the AVM+WM task involved a memory component. It may result in a more engaged state with enhanced attentional demands, leading to a relatively modest increase in the planning and movement time after administration of 0.8 mg/kg ketamine. This interpretation is also supported by the findings regarding the level of task engagement (see Fig. 9), which showed the tendency of task engagement was adversely affected by 0.8 mg/kg ketamine in the AVM-NC tasks, but not in the AVM+WM task, suggesting a more engaged state during the AVM+WM task. Taken together, these results suggest that ketamine may not substantially impair attention during more complex tasks, particularly those involving working memory.

Additionally, we observed a negative correlation between reaction time and accuracy after ketamine injection, indicating that the effects of ketamine vary on a trial-by-trial basis, and that there may be a common mechanism underlying this variation, affecting both the quality (as reflected by reduced accuracy) and the speed (as reflected by prolonged reaction time). Our findings suggest that the prolonged reaction time, which is easy to measure in a clinical setting, can serve as an effective indicator of the ketamine effects on higher cognition.

As a study investigating the effects of ketamine on behavior, our results provide important insights for further understanding the underlying neural mechanisms. We examined the nonspecific effects of ketamine on motivation by calculating the level of engagement of the subjects, as reflected by the ratio of completed trials to the total number of trials. To exclude such nonspecific effects, we reported the accuracy and reaction time results in the main text based on the data of completed trials only. Our findings suggest that ketamine tends to lower the level of engagement of animals in the AVM-NC task with a statistically significant difference and in the AVM task with a downward trend, while the level of engagement in the AVM+WM task was not affected by ketamine (Fig. 8). One possible explanation is that low-dose ketamine induces decreased attention in AVM, which do not require delayed memory.

Previous research has shown that ketamine (0.3 mg/kg) caused attention deficits in humans, which can be detected using electroencephalogram and horizontal electrooculogram (Oranje et al., 2000). Similarly, Umbricht et al. (2000) found that ketamine induced deficits in auditory and visual attention. Additionally, it has been suggested that ketamine may have a stronger effect on top-down attentional processing, i.e., voluntary guidance of attention by internal goals, rather than bottom-up attentional capture (Fuchs et al., 2015), i.e., the involuntary capture of attention by prominent environmental events, which is consistent with literature indicating that ketamine can affect attention-related EEG components (Schwertner et al., 2018; Thiebes et al., 2018). Deficits in top-down attention could explain the lowered accuracy, prolonged planning time, and difficulty in acquiring new mapping rules, as these functions rely on proper guidance of attention by internal goals. However, during the experiment, the macaques in the delayed memory stage of WM had a bottom-up attention that might not have been significantly disturbed by low doses of ketamine. Future studies are needed to determine how low-dose ketamine impairs top-down and bottom-up attention and how this effect may lead to profound deficits in AVM and WM.

An interesting result obtained in our study is that ketamine significantly induced more incorrect responses when the visual cue is changed from the previous trial in each task. This result is consistent with prior findings that PFC injury or lesion can impair behavioral performance in an AVM+WM task, particularly when visual stimuli change from the previous trials (Diamond and Goldman-Rakic, 1989; Rowe et al., 2007). However, we also observed that this specificity in errors also holds for the AVM task and AVM-NC task, which do not involve a delay period for maintaining temporal memory. Additionally, our findings showed that the wrong response induced by ketamine was observed in cue unchanged trials, although not significantly. If memory maintenance were disrupted, the performance of cue unchanged trials would be the same as cue changed trials. Thus, we inferred that the impaired ability to make rule-based decisions, rather than impaired WM maintenance, may be the primary cause of incorrect choices after administration of low-dose ketamine.

To investigate the effect of ketamine on perseveration, a phenomenon similar to performance after PFC injury (Butter, 1969; Iversen and Mishkin, 1970) and chronical cocaine administration (Jentsch et al., 2002), we analyzed the percentage of incorrect trials across three trial-forms (AB, AAB, and AAAB) under 0.8 mg/kg ketamine condition. Our results showed no significant differences between the three task forms (see Extended Data Fig. 3-2). If low-dose ketamine primarily affects perseveration, the percentage of incorrect trials in the AAB or AAAB form would have been higher than that in the AB form. Our findings suggest that ketamine mainly induced more incorrect responses because of trial-switch rather than perseveration.

According to these results, we can infer that low-dose ketamine mainly impacts the brain’s ability to flexibly coordinate decision-making and actions to achieve the current goal (Miller and Cohen, 2001; Fuster and Bressler, 2015), as well as represent the information regarding rules on how to reach the goal (Buckley et al., 2009; Saez et al., 2015; Duverne and Koechlin, 2017). This raises the possibility that the NMDA receptor is involved in the response to a choice stimulus and evolves from a stimulus-specific population response to a variety of final decision-related states during decision-making. It is consistent with characteristics of dynamic coding in the prefrontal cortex that can adapt to changes in behavioral context (Stokes et al., 2013). By blocking NMDAR, ketamine may disturb the capabilities of the PFC in goal-related planning, especially when the situation changes rapidly, e.g., when the visual cues change from trial to trial. This is in line with previous studies showing that low-dose ketamine can reduce NMDA-mediated frontal-to-parietal connectivity (Muthukumaraswamy et al., 2015) and alter oscillations of lateral PFC in rule-based tasks (Ma et al., 2018). However, the exact mechanism underlying the specificity of ketamine-induced errors requires future studies that combine behavioral assessments and electrophysiological recordings to delineate.

## Synthesis

Reviewing Editor: Niko Busch, Westfalische Wilhelms-Universitat Munster Decisions are customarily a result of the Reviewing Editor and the peer reviewers coming together and discussing their recommendations until a consensus is reached. When revisions are invited, a fact-based synthesis statement explaining their decision and outlining what is needed to prepare a revision will be listed below. The following reviewer(s) agreed to reveal their identity: Ryo Sawagashira.

Synthesis

Thank you for submitting the revised version of your manuscript. The reviewers and I have evaluated your manuscript once more, and I am happy to let you know that we find the manuscript suitable for publication once a couple of minor comments are addressed, specifically the limitations and corrections mentioned by reviewer 2.

Reviewer 1

This resubmitted manuscript is well revised and greatly improved. Unfortunately, eye movement data were not recorded, but this is well compensated for by the ERP results. Therefore, this manuscript is suitable for acceptance.

Reviewer 2

The authors did well in addressing all of my concerns.

I have 2 minor comments: 1. Regarding my concern #1 (which was also raised by the other reviewer), the ERP analysis shows only minor changes within the first 70ms and we could assume that the animals recognized the visual stimuli. But since the ERPs were not from visual areas, I suggest that the authors mention the lack of eye/video tracking/visual ERP as a limitation to this study. 2. There are still a few uses of ‘correct percent’ and ‘wrong perceptage’, which I hope the authors could correct.

## Author Response

Reviewer 1

In this manuscript, the authors investigated the effects of subanesthetic dose of ketamine on well-trained and novel learning, and working memory behaviors using macaque monkeys. The authors demonstrated that ketamine diminished behavior for all three conditions: AVM, AVM-WM, and AVM-NC. Moreover, they evaluated the changes in response latency, then concluded that ketamine’s effect is largely on planning time rather than movement time.

This paper was well written and will become a good reference for how subanesthetic dose of ketamine affects on associative learning. Here is specific comments and several minor points. I hope these comments help the authors to improve this work.

Reply：

We appreciate the reviewer’s positive evaluation of the paper and very helpful comments/suggestions. Please see below for our point-by-point reply.

Specific comments

1) In Figure 2c, with late-post injection, the accuracy appears to be rather elevated at 0.8 mg/kg dose of ketamine. Please consider the same analysis in the late-post injection window as the one shown in the inset (early phase). Related to this, why does the time course of accuracy in the control and 0.2 mg/kg conditions appear to be below 50% (below chance level) in later phase?

Reply：

According to the suggestion, we have conducted the same analysis for the data collected during the late-post injection period. We found that there were no significant differences among the saline, 0.2 mg/kg ketamine, and 0.8 mg/kg ketamine conditions during the late-post injection period in the AVM task for both monkeys (monkey A, K-W test, p = 0.76; monkey Z, p = 0.98). 2

Similarly, there were no significant difference between these three conditions during the late-post injection period in the AVM+WM task (monkey A, p = 0.85; monkey Z, p = 0.13). These results were added to the results section of revised paper.

Regarding the accuracy in the control and 0.2 mg/kg conditions appear to be below 50%, we found that this was due to an error that these accuracies were calculated by the number of correct trials divided by the number of all trails, rather than the number of completed trials. In the late post-injection period, the lowered motivation caused more incomplete trials, leading to the apparent accuracy lower than 0.5. We apologize for this error, and the figure has been updated to reflect the accuracy among all completed trials, as we did for all other analyses in the paper.

2) In Figure 2a, is it possible that ketamine deteriorated animals’ gaze on the visual stimuli? If the monkeys are not able to see the monitor properly, it seems difficult to conclude that ketamine affected associative learning. Did you record eye movement data? If not, do you have any suggestions from the analysis of other errors (e.g., percentage of time-out error or premature response)?

Reply：

Thank you for this important comment, which is shared by both reviewers and the editor. Unfortunately, no eye movement data were recorded during this experiment. However, we recorded electrophysiological signals by chronically implanted microelectrode arrays in the prefrontal cortex (PFC), dorsal premotor cortex (PMd) and the hippocampus in both animals, which we analyzed to address this concern.

Specifically, we analyzed the event-related potentials (ERPs) in the PFC and PMd using electrophysiological signals recorded in one channel during the cue phase of the AVM task under the 0.8mg/kg ketamine condition. To 3 address the concern that the error trials may be caused by the animal not being able to see the cue properly, we specifically compared the ERPs of all incorrect trials among the pre-, early-, and late-post-injection phases. We found that in both the PFC and the PMd, EPRs calculated based on data collected among these three phases were highly similar (Fig. R1), indicating similar early visual processing.

Figure R1. The ERPs in response to the visual cue during the AVM task. The ERPs were obtained by averaging local field potentials recorded by one electrode in either the PFC or the PMd array across all incorrect trials.

These results suggest that 0.8mg/kg ketamine did not affect the monkeys’ ability to attend or to see the cue. We have now mentioned these control analyses in the discussion section in Page 17 of the revised paper. However, in the current paper we focus on analyzing the behavioral performance, so we plan to include more comprehensive electrophysiological analyses in subsequent articles.

4

3) I understand that the effect of ketamine is less likely to occur if the previous trial is “same”. Is it correct to understand that it is perseveration? If possible, could you analyze not only previous trial but also two or three previous trials?

Reply：

Thanks for the helpful suggestions. Accordingly, we have extended the analysis to include two and three previous trials. To facilitate comprehension, we note the same cue and different cue situations as AA and AB, respectively.

The first letter denotes the cue for the previous trial, while the second letter represents the cue for the current/analyzed trial. If the previous two/three trials were considered, we can similarly define the same cue situation (AAA/AAAA) and the different cue situations (AAB/AAAB). We added the above content to the revised paper (Page 8).

To address the possibility of “perseveration,” in the one previous trial situation (AA or AB) analyses we now only included the cases in which the previous trial was associated with the correct response (the results shown in the original Figure 3 did not impose this criterion). We obtained nearly identical results compared to the original analyses, which are shown in Figure R2. In the AB situation, we found that 0.8 mg/kg ketamine resulted in significantly more errors than the saline condition in the AVM task (K-W test, p = 0.0006, post hoc, p = 0.0009), AVM-NC task (K-W test, p = 0.0017, post hoc, p = 0.0020), and AVM+WM task (K-W test, p = 0.0273, post hoc, p = 0.0252). Additionally, we observed that 0.8 mg/kg ketamine induced significantly more error trials than the 0.2 mg/kg ketamine condition in the AVM task (K-W test, p = 0.0008, post hoc, p = 0.0222) and AVM-NC task (K-W test, p = 0.0017, post hoc, p = 0.0104). However, we found no significant difference between the saline and 0.2 mg/kg ketamine conditions in each task. In the same cue (AA) situation, there was no significant difference among saline, 0.2 mg/kg, and 0.8 mg/kg ketamine conditions in all tasks.

5

Figure R2 has also been included as Figure 3 in the revised text.

Figure R2. The percentage of incorrect trials in the same cue and different cue situations during a 60-min period after the injection. The average wrong percent of trials after injection for the saline group (Nacl), 0.2 mg/kg ketamine group (Ket 0.2), and ketamine 0.8 mg/kg group (Ket 0.8) are shown. All error bars are SEM. *<0.05, **<0.01, ***<0.001.

To further investigate the effect of perseveration on the results, we compared the percentage of incorrect trials across the conditions of AB, AAB, and AAAB under the 0.8 mg/kg ketamine condition (Figure R3). Our results indicated no significant difference among these conditions with 0.8 mg/kg ketamine in all three tasks. This suggests that 0.8 mg/kg ketamine mainly increased the percentage of incorrect responses when the current trial switched from the previous one and was not specifically related to “perseveration.” Because if it was perseveration, the accuracy of AAB or AAAB conditions would be lower than that of the AB condition. We added these results in the revised paper (Page 12). The Figure R3 has also been included as Figure 3-2 in the revised text. Figure R3. The percentage of incorrect trials across three conditions (AB, AAB, and AAAB) for each task with 0.8 mg/kg ketamine. The error bars correspond to the standard

6 error of the mean (SEM).

4) In Figure 6, correct rates in slower RT trials are always lower than that in fast RT trials in all three conditions. From this, the authors concluded that reaction time and accuracy were negatively correlated. Did you try any correlation analysis for this point?

Reply：

Thanks for the suggestion. We analyzed the correlation between the accuracy and the reaction time (RT) of trials in the early post-injection period under the 0.8mg/kg ketamine condition.

Based on our analysis of planning and movement time (as demonstrated in Figure 4), we selected trials with the RT less than 2.5 s for AVM and AVM+WM tasks and those with the RT less than 2.0 s for AVM-NC task. We then analyzed the correlation between the RT and the accuracy separately for each task. We subdivided the reaction time range (0 - 2.5 s or 0 - 2.0 s) using a 0.05 s time window and calculated the mean accuracy in each window. We then obtained the correlation coefficient between the accuracy and the RT .

Our results demonstrate a negative correlation between the RT and the accuracy after administrating 0.8mg/kg ketamine in all three tasks (as shown in Figure R4). The correlation coefficient was -0.4576 for the AVM task (p<0.001), -0.6016 for the AVM-NC task (p<0.001), and -0.4257 for the AVM+WM task (p<0.001). We added these results in the revised paper (Page 15). Figure R4 has also been included as Figure 8 in the revised paper.

7

Figure R4. The correlation between the accuracy and the reaction time after administrating 0.8 mg/kg ketamine. The blue dots in the figure represent the corresponding average accuracy within the corresponding range of the reaction time.

Minor points

1) In Introduction section, the abbreviation of associative learning is written “AVM”. Is it correct?

Reply: This is a typo. AVM should be AL. It is corrected now. Thanks for pointing this out to us. (see Page 2)

2) In the last part of Results section, what is “NPAL task”? Is it “AVM-NC”?

Reply: Yes. It should be AVM-NC. Thanks for pointing this out. (see Page 16)

3) In 2nd point in General Discussion section, “prolonged reaction time, but with a stronger impact on the planning time than the movement time.” seems to be limited to NC task.

Reply: We agree and have made revisions accordingly. We have renamed the section to “Differential effects of ketamine on planning and movement during the response stage of the early post-injection period” and revised the following statement at the end of the section: “These findings suggest that ketamine prolongs reaction times by interfering with both planning and movement, with planning being more severely affected, particularly in the AVM-NC task, which involves learning novel visual-motor mapping rules.” (see Page12-13) 8

4) In the legend of Figure 3, “percept” should be changed into “percent”.

Reply: Corrected. Thanks.

9

Reviewer 2

Effects of ketamine on cognitive and motor processes are of interest given its increasingly wide usage as a fast-acting antidepressant. The authors examined and reported deleterious effects of acute low dose ketamine on different aspects of arbitrary visuomotor mapping including learning, motor execution and working memory in macaque monkeys. The study is interesting and potentially of interest to a wide audience of researchers. However, due to problems in the choice of statistical analysis and interpretation of results, the manuscript needs major revisions before it could be accepted for publication.

The writing per se also requires many minor revisions to improve readability and could benefit from a professional editing service.

Reply：

We appreciate the reviewer’s thorough evaluation and constructive suggestions. Please see below for our point-by-point reply.

Major points:

1. A major shortcoming/future direction of the study is the absence of gaze analysis, which is possible to do in head-free animals. As a result, we remain unclear whether the animals’ impairment was in the recognition of the cues, the retrieval of the stimulus-target mapping, or the guidance of the mapping over choice behaviour. All these types of impairment are possible, given how ketamine gives healthy volunteers a full set of positive, negative and cognitive symptoms resembling those seen in schizophrenia. One thing the authors could do (if the trained monkeys are still available), is to shorten the cue period in the AVM task and see if this poses extra challenge (more errors/longer RT) when they’re treated with ketamine. If so, this would indicate that visual recognition is also impaired by ketamine in monkeys. 10

Reply：

Thank you for your insightful comments. We have tried to collect more data, including the eye movement signals and the performance with shortened presentation of the cue. However, the reported results were obtained some time ago, and now the head post in both monkeys become unstable, preventing successful new data collection. Fortunately, we were able to address the concern that the animal may not see the cue properly by analyzing ERPs recorded during the experiments. Please see our reply to the 2nd question of the Reviewer #1, which suggest that ketamine did not affect the monkeys’ ability to see the cue.

2. The authors mentioned ‘trial done percentage’ in Methods but never showed relevant results. This would be necessary to show if ketamine had any effect on the task-engagement of the monkeys. Could they please add analyses and figures on this?

Reply：

Thank you for the question. In the original manuscript, such results were presented in Supplementary Figure 6, which demonstrates a significant decrease in trial done percentage in AVM-NC at 0.8mg/kg ketamine, and a downward trend in AVM tasks, but no apparent effect on trial done percentage in AVM+WM tasks. To make this point clearer, we included the Supplementary Figure 6 as Figure 9 in the revised paper, and mentioned these results in Page 15-16.

3. Many sessions with ketamine on board were collected, allowing the analysis of any potential chronic effect of ketamine. This could be detectable even though the short-term effect disappears after 1hr. Could the authors please compare the sessions collected early vs later in the study to see if 11 ketamine has a long-term impact on cognition/motor control?

Reply：

We appreciate your suggestion to investigate the long-term effects of ketamine. Accordingly, we compared the sessions collected early in the study to those collected later in the study (separated by 15 days in the AVM and AVM+WM tasks, and 14.3 days in the AVM-NC tasks) to evaluate whether ketamine has any long-term impact on cognition and motor control.

We analyzed the performance of the pre-injection phase, including accuracy, planning time, movement time, and trial done percentage in the AVM and AVM+WM tasks. While there was no pre-injection phase in the AVM-NC task, we analyzed the sessions under saline conditions and examine the potential long-term effect. We took the mean values of each session, and used estimation statistics (Ho et al., 2019) to examine the differences between three early and three late sessions. Our findings showed that during the AVM-NC task, planning time in the late sessions was significantly higher than in the early sessions (see Figure R5). The mean difference in planning time between the early and late sessions for both monkeys was 0.186 s (95% CI=0.066 to 0.335 s, p=0.033).

Similar trends can be observed in individual animals, where monkey A’s mean difference between the early and late sessions was 0.199 s (95% CI=0.042 to 0.375 s, p=0.092), and monkey Z’s mean difference was 0.173 s (95% CI=0.108 to 0.257 s, p=0.065). However, we observed no significant changes in accuracy, movement time, and percentage trial done after ∼2 weeks of intermittent ketamine administration (with at least a 48-hour interval between them). In addition, we observed no significant differences in the accuracy, planning time, movement time, and trial done percentage in the AVM and AVM+WM tasks. Thus, in our results, ketamine exhibited no detectable chronical effects except for the planning time in the AVM-NC task. We added these results in the revised text in Page 14. Figure R5 has also 12 been included as Figure 6-2 in the revised text. Figure R5. The planning time of early and late saline sessions in the AVM-NC task. The mean interval time between early and late sessions was 14.3 days. a. The mean planning time of both monkeys between early (blue) and late (orange) sessions. b. and c.

The performance of monkey A and monkey Z.

We note that 2 weeks may not be sufficient to fully explain the chronical effects of low-dose ketamine on cognition/motor control. Although we did not intend to investigate the chronic effects of low-dose ketamine in our experiment, the results may still provide valuable insights for researchers who are interested in it. We appreciate your suggestion and will conduct additional analyses to explore this question further in the future.

4. Planning and movement times were prolonged to a lesser extent in the WM task, compared to the AVM and AVM-NC tasks. Was this effect significant? If so, could the authors please discuss this effect? This effect contrasted with the finding that the performance suffered more in the WM task, which is very interesting.

Reply：

Thanks for the question. We have conducted further analyses to examine the significance of the effect in prolonging planning and movement times in the WM task, compared to the AVM and AVM-NC tasks. We analyzed the prolonged part of planning time and movement time with 0.8mg/kg ketamine and performed statistical analysis across the three tasks. Specifically, we 13 calculated the average planning time and movement time of each session in the early post-injection period and obtained the prolonged change relative to saline conditions by subtracting the values with 0.8mg/kg ketamine by the mean values of all saline conditions. We then used ANOVA or Kruskal-Wallis (K-W) test (if the data did not conform to the normal distribution) to examine the statistical significance. The results showed that there was no significant difference across the three tasks in both monkeys in planning time (ANOVA, F (2, 28) = 1.450, p = 0.2516) and movement time (K-W test, p= 0.3506), as illustrated in the Figure R6. However, under the 0.8mg/kg ketamine condition, the prolonged change in planning and movement time for the AVM+WM task indeed showed a downward trend compared to the AVM and AVM-NC tasks.

We added these results in revised text in Page 13-14. Figure R6 has also been included as Figure 6 in the revised paper. Figure R6. The change of planning time and movement time under 0.8mg/kg ketamine conditions relative to the saline condition. The dots indicate the change in planning/movement time relative to saline at a dose of 0.8mg/kg during the early post-injection phase of the task. One possible explanation for this phenomenon is that task difficulty is a contributing factor. Specifically, the AVM+WM task involved a memory component. It may result in a more engaged state with enhanced attentional demands, leading to a relatively modest increase in the planning and movement time after administration of 0.8 mg/kg ketamine. This interpretation 14 is also supported by the findings regarding the level of task engagement（see Fig. 9）, which showed the tendency of task engagement to adversely affected by 0.8 mg/kg ketamine in the AVM and AVM-NC tasks, but not in the AVM+WM task, suggesting a more engaged state during the AVM+WM task. Taken together, these results suggest that ketamine may not substantially impair attention during more complex tasks, particularly those involving working memory. We added the above discussion to Page 19 in the revised text.

5. The result section named “Ketamine affects planning more efficiently than movement at the response stage of the early post-injection period”: it is strange to call a negative impact ‘more efficient’. Critically, since only one task showed a significant difference in movement and planning time, this claim (the section title) is not broadly justified. It only works with a specific reference to the AVM-NC task.

Reply：

We have renamed the section to “Differential effects of ketamine on planning and movement during the response stage of the early post-injection period” and revised the following statement at the end of the section: “These findings suggest that ketamine prolongs reaction times by interfering with both planning and movement, with planning being more severely affected, particularly in the AVM-NC task, which involves learning novel visual-motor mapping rules.” (see Page 12-13)

6. Also, about Figure 5 and related text: it seems that 3 paired-t tests were done for the 3 tasks. This is not valid. Was the p-value reported corrected for family-wise error? In any case, a mixed-model ANOVA (injection as within and task as between-subject variables) is the appropriate choice. It will likely boost 15 (not undermine) the statistic power. Similarly, in 1st paragraph of Discussion: point 2 is not justified except in AVM-NC.

Reply：

Followed the suggestion, we used the Benjamini-Hochberg method to correct for multiple comparisons on the three p-values (0.0759,0.0003 and 0.0655) obtained from the tests. This resulted in corrected p-values of 0.1392, 0.0017, and 0.1392 for the AVM, AVM-NC, and AVM+WM tasks, respectively. And we revised the corrected p-values in the text in Page13 as “(paired t-test, t = 4.299, df = 12, two-tailed, p = 0.0017)”. As mentioned above in our reply to the question 5, we also conducted ANOVA, to examine the differences on prolonged planning and movement time among the three tasks.

We appreciate your thorough review of our manuscript and thank you for your constructive comments. Your feedback has helped us improve the paper significantly.

7. 1st paragraph of Discussion: point 3 is not justified. The authors found that trials with longer RTs were less accurate, not vice versa (i.e. they did not group trials based on accuracy).

Reply：

Thanks for the question. This has also been raised by the Reviewer #1.

We have conducted further analysis on correlation between the accuracy and the reaction time (RT) of trials in the early post-injection under the 0.8mg/kg ketamine condition. Our results demonstrate a negative correlation between reaction time and accuracy in the early post-injection under the 0.8mg/kg ketamine condition for all tasks (as shown in Figure 8). Please see our reply to question 4 of Reviewer #1 for details. 16

8. In ‘significance statement’ and again in Discussion: we can’t say that what’s impaired by ketamine necessarily rely on NMDA transmission, since it is such a ‘dirty’ drug and interacts with dopamine D2 and many other receptors. Such statement can only be reserved for highly selective compounds. Instead, one could say, one possible explanation to the negative impact on learning/performance is through NMDA receptor blockade. As for WM, studies have done and shown that a metabolite of ketamine led to increased glutamate release (Yang et al 2018, last author Hashimoto), increasing the level of activity in neurons (many studies), leading to higher noise and impaired signal (e.g. Ma et al 2015, last author Everling). The authors need to do a more thorough examination of the existing literature and cite more relevant studies.

Reply：

Thank you for pointing this out. We have carefully considered your suggestions and revised the Significance Statement as “This study found that acute low-dose ketamine significantly impairs the ability of arbitrary visual motor mapping, a critical form of associative learning for flexible behavior, including stimulus-response contingency learning or learning of new contingencies. We conducted a thorough analysis and identified intrinsic features of ketamine-induced functional deficits, including decreased accuracy, prolonged planning and motor execution time, increased error tendency when visual cues changed from trial to trial, and stronger impact on associative learning and another key higher-order cognitive function, working memory. These findings contribute to a better understanding of the potential acute cognitive side effects of low-dose ketamine and promote its safe use in medical practice.”.

We also have added the following to the discussion section in Page 17- 18: “ One possible explanation for the negative impact on learning/performance is through NMDA receptor blockade. However, ketamine 17 may also exert its effects through other mechanisms. For example, ketamine can activate the GABAergic system (Zhang et al., 2021) and increase dopamine release (Kokkinou et al., 2018). As for WM, studies have done and shown that a metabolite of ketamine led to activation of rapamycin (mTOR) signaling (Yang et al., 2018). In addition, ketamine can increase the level of activity in neurons (Roussy et al., 2021, Ma et al., 2018, Jang & MacIver, 2021, Wang et al., 2013), leading to increased noise and impaired signal (Ma et al., 2015). “

Minor points:

1. Please insert page numbers. Right now, it’s nearly impossible to discuss the details in the text.

Reply: Thank you for your comment. We apologize for the inconvenience caused by the lack of page numbers. We have now added them to facilitate discussion of the text.

2. Please verify citations to ensure that the description of the cited studies are accurate.

Reply: We have double-checked all the citations and made sure that the descriptions of the cited studies are accurate.

3. Please add the duration of each epoch to Fig 1a, b to facilitate reading.

Reply: We have added the duration of each epoch to Fig 1a, b to make it easier to read.

4. Citation style is inconsistent throughout the manuscript and does not conform to eNeuro’s requirements. Please update the style before submission/re-submission to a different journal.

18

Reply: We have updated the citation style to conform to eNeuro’s requirements before resubmitting.

5. There are many wrong uses of phrases in the manuscript, e.g. correct percent percentage correct, etc. Please consider using a professional editing service.

Reply: Thank you for your feedback. We have used a professional editing service to correct the whole manuscript.

6. ‘Wrong trial percent’ ‘percentage incorrect’ or ‘percentage error’, please change this in Fig 3 y-axis label too.

Reply: Thank you for your comment. We have changed the y-axis label in Fig 3 from ‘Wrong trial percent’ to ‘Percentage incorrect’ to make it clearer. In addition, we also changed the y-axis label in Figures.

7. There are many typos throughout the manuscript, e.g. trail-trial, percept-percent, severally-severely, etc. Please read carefully and revise.

Reply: Thank you for pointing this out. We have carefully read through the manuscript and corrected the typos.

8. In Introduction: ‘a wildly used’ ‘widely used’

Reply: We have corrected the mistake and changed ‘a wildly used’ to ‘widely used’ in the Introduction.

9. Top of 2nd page in Intro: ‘Associative learning (AVM)’-I thought AVM referred to arbitrary visuomotor mapping?

19

Reply: Thank you for your question. We apologize for the confusion caused by the use of AVM. You are correct that AVM refers to arbitrary visuomotor mapping, not associative learning. We have revised the text accordingly in page 2.

10. Also: ‘Arbitrary visuomotor mapping (AVM) is a specific form of AVM’-this doesn’t make sense. Please refrain from using abbreviations when it involves 2 words, as per SfN journals’ requirement.

Reply: Sorry. This is a typo. We have modified the text as “Arbitrary visuomotor mapping (AVM) is a specific form of associative learning” in Page 2-3.

11. In Methods: ‘at least 1 day separation’, does this mean ∼48hrs between ketamine injections as the shortest interval, or 24hrs?

Reply: Thank you for your question. We meant at least a 48hrs separation between ketamine injections, so we have clarified this in the Methods section on page 6. 12. Fig 4: please make sure the insets do not overlap with the main plot, like in 4a, c and e.

Reply: We have revised the figure to ensure that the insets in Fig 4a, c and e do not overlap with the main plot.
