# Supplementary figures and images for: Low-Dose Ketamine-Induced Deficits in Arbitrary Visuomotor Mapping in Monkeys
Source: eNeuro. 2023 Jun 23;10(6):ENEURO.0015-23.2023. doi: 10.1523/ENEURO.0015-23.2023 (PMC10309660; doi:10.1523/ENEURO.0015-23.2023)

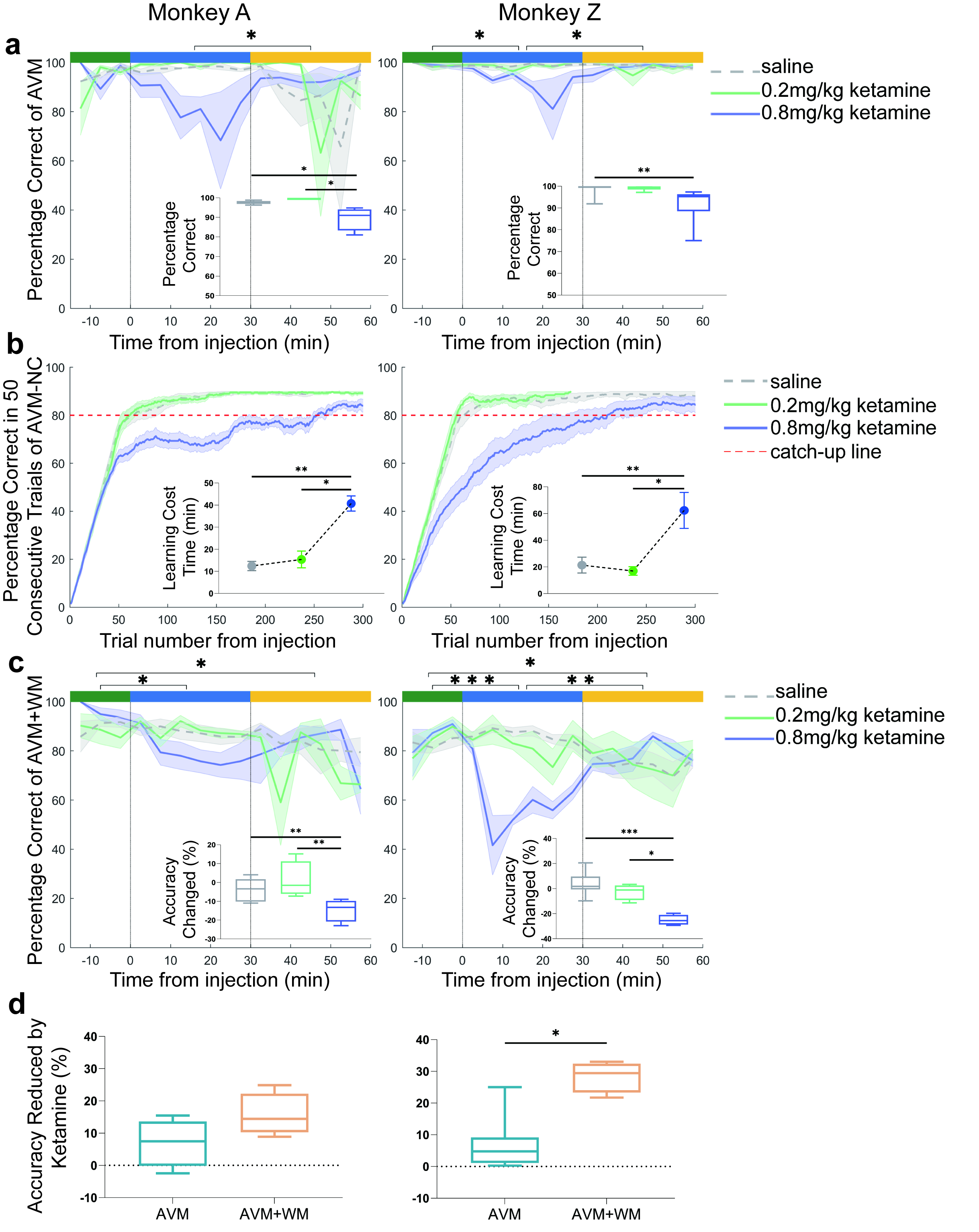

Supplement: Extended Data Figure 2-1 — Behavioral performance per subject. a, Percentage of correct trials in the AVM task for saline (gray), ketamine 0.2 mg/kg (green), and ketamine 0.8 mg/kg (blue) sessions. The lower right corner box plot shows the average percentage of correct trials during the early-postinjection period across three conditions. b, Accuracy in 50 consecutive trials in the AVM-NC task for saline, ketamine 0.2 mg/kg, and ketamine 0.8 mg/kg sessions. The lower right corner plot shows the learning cost time across three conditions. c, Percentage of correct trials in the AVM+WM task for saline, ketamine 0.2 mg/kg, and ketamine 0.8 mg/kg sessions. The lower right corner box plot shows the accuracy changed from the preinjection period to early-postinjection period across three conditions. d, Comparison of the effects of ketamine on AVM and AVM+WM tasks for Monkey A and Monkey Z. The effect of 0.8 mg/kg ketamine was quantified as the percent performance drop, calculated by (Apre − Aearly-post)/Apre %, where Apre and Aearly-post are the accuracy of the preinjection period and early-postinjection period, respectively. The bars represent the declined rate of accuracy. All error bars represent SEM. Statistical significance is denoted by *p < 0.05, **p < 0.01, ***p < 0.001. Download Figure 2-1, TIF file. [file enu-eN-NWR-0015-23-s02.tif]

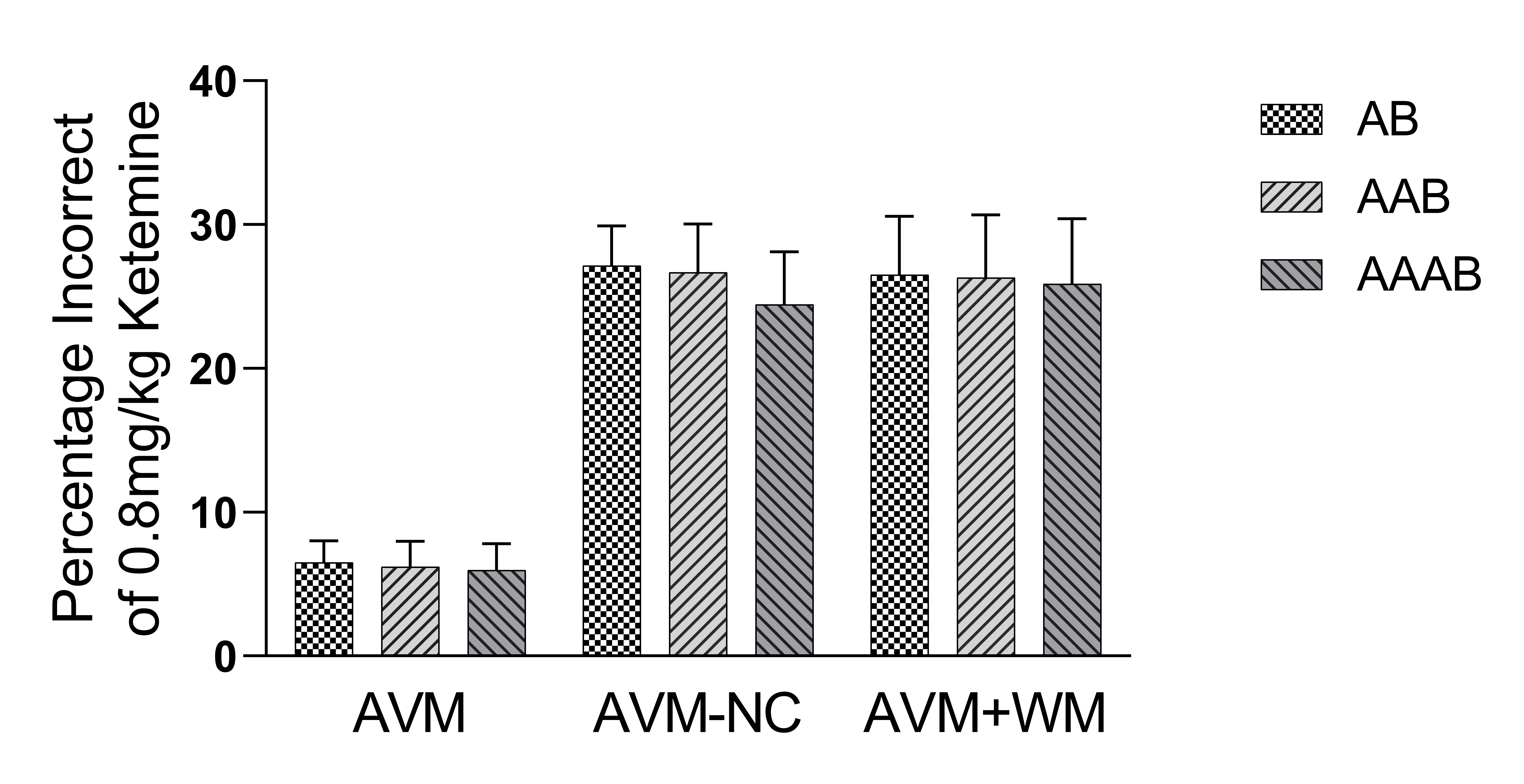

Supplement: Extended Data Figure 3-2 — The figure depicts the percentage of incorrect trials across three trial-forms (AB, AAB, and AAAB) for each task under the 0.8 mg/kg ketamine condition. In the AAB and AAAB trial-forms, the cue from the previous trials that had been responded correctly was denoted as A, while the cue from the analyzed trial was denoted as B. The error bars correspond to the SEM. Download Figure 3-2, TIF file. [file enu-eN-NWR-0015-23-s04.tif]

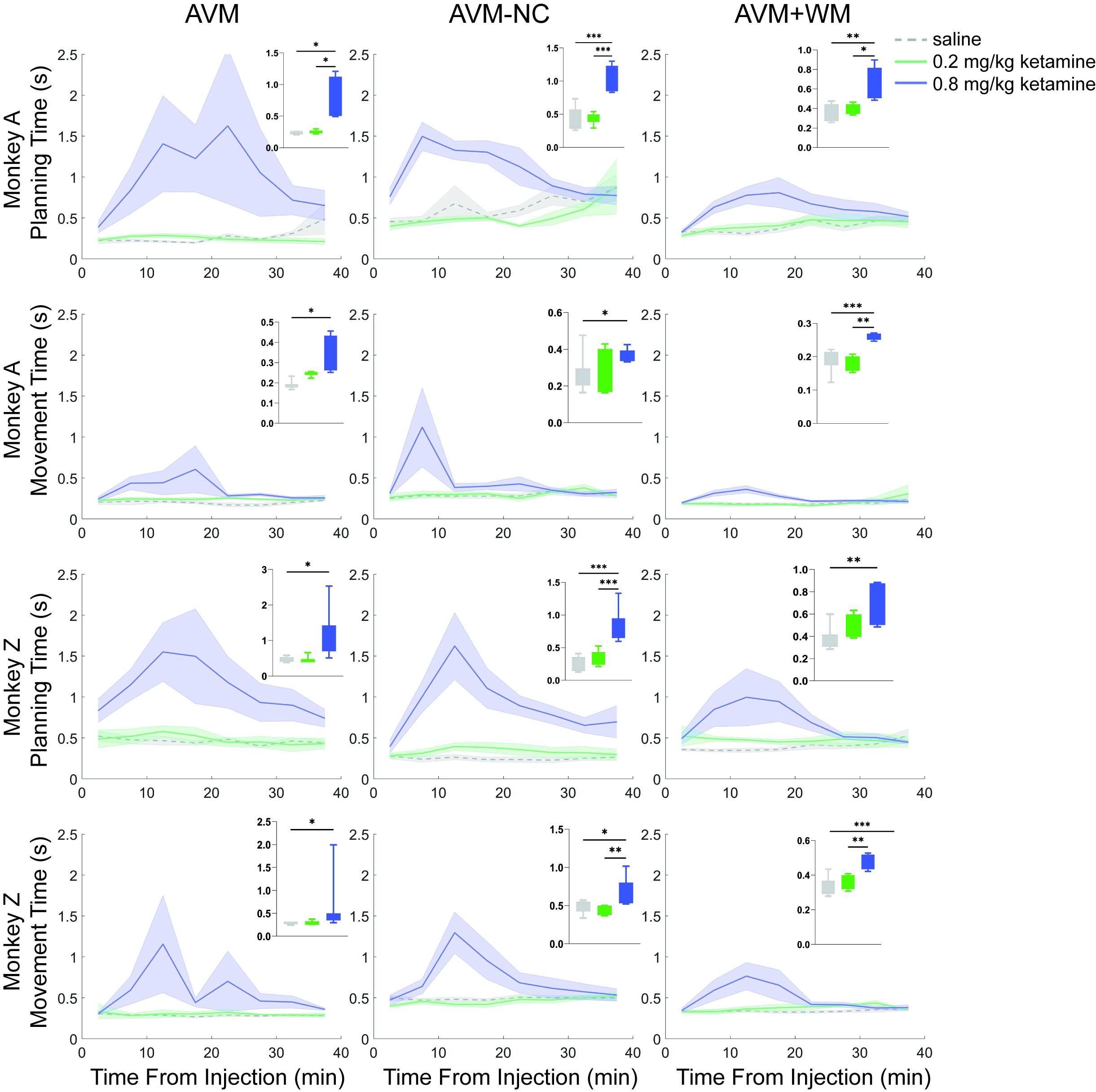

Supplement: Extended Data Figure 4-1 — Planning and movement time in early-postinjection per subject. Planning time and movement time performance of early-postinjection period in AVM, AVM-NC and AVM+WM task over saline (gray), 0.2 mg/kg ketamine (green) and 0.8 mg/kg ketamine (blue) conditions for Monkey A and Monkey Z. The higher right corner box plot was the average planning time or movement time in the early-postinjection period over saline (gray), 0.2 mg/kg ketamine (green) and 0.8 mg/kg ketamine (blue) conditions. All error bars are SEM. *p < 0.05, **p < 0.001, ***p < 0.001. Download Figure 4-1, TIF file. [file enu-eN-NWR-0015-23-s05.tif]

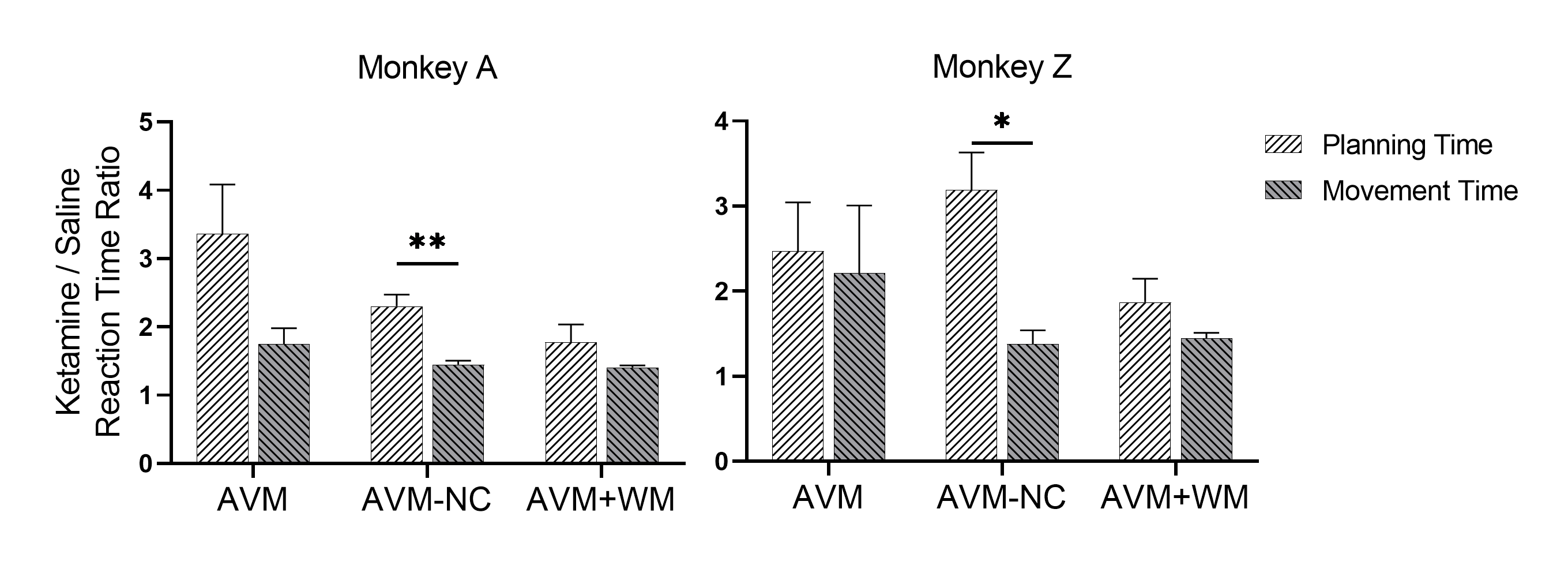

Supplement: Extended Data Figure 5-1 — Comparison of planning time and movement time during the early-postinjection period of each task per subject. To assess the effects of 0.8 mg/kg ketamine on planning time and movement time, we calculated the prolonged index for all each task. The prolonged index is defined as the ratio of Tplan-0.8/Tplan-saline and Tmove-0.8/Tmove-saline, where Tplan-0.8 (Tmove-0.8) and Tplan-saline (Tmove-0.8) denotes the mean planning (movement) time under the 0.8 mg/kg ketamine and saline conditions, respectively. All error bars are SEM. Statistical significance is denoted by *p < 0.05, **p < 0.01. Download Figure 5-1, TIF file. [file enu-eN-NWR-0015-23-s06.tif]

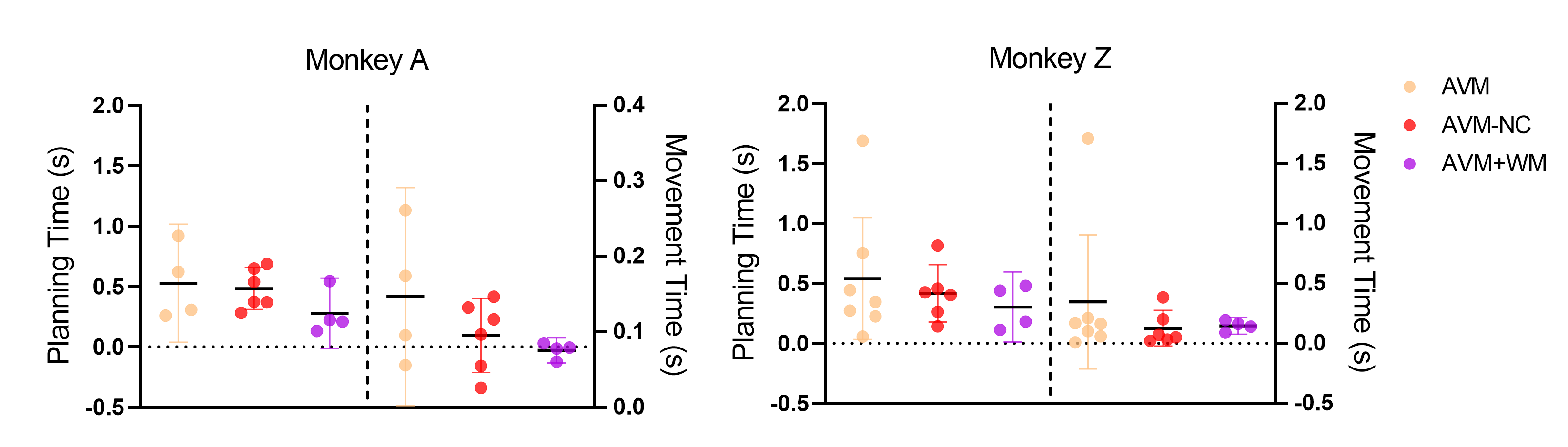

Supplement: Extended Data Figure 6-1 — The prolonged change of planning time and movement time under 0.8 mg/kg ketamine conditions relative to saline conditions. The dots in the figure indicate the change in planning (movement) time relative to saline at a dose of 0.8 mg/kg during the early-postinjection phase of the task. Download Figure 6-1, TIF file. [file enu-eN-NWR-0015-23-s07.tif]

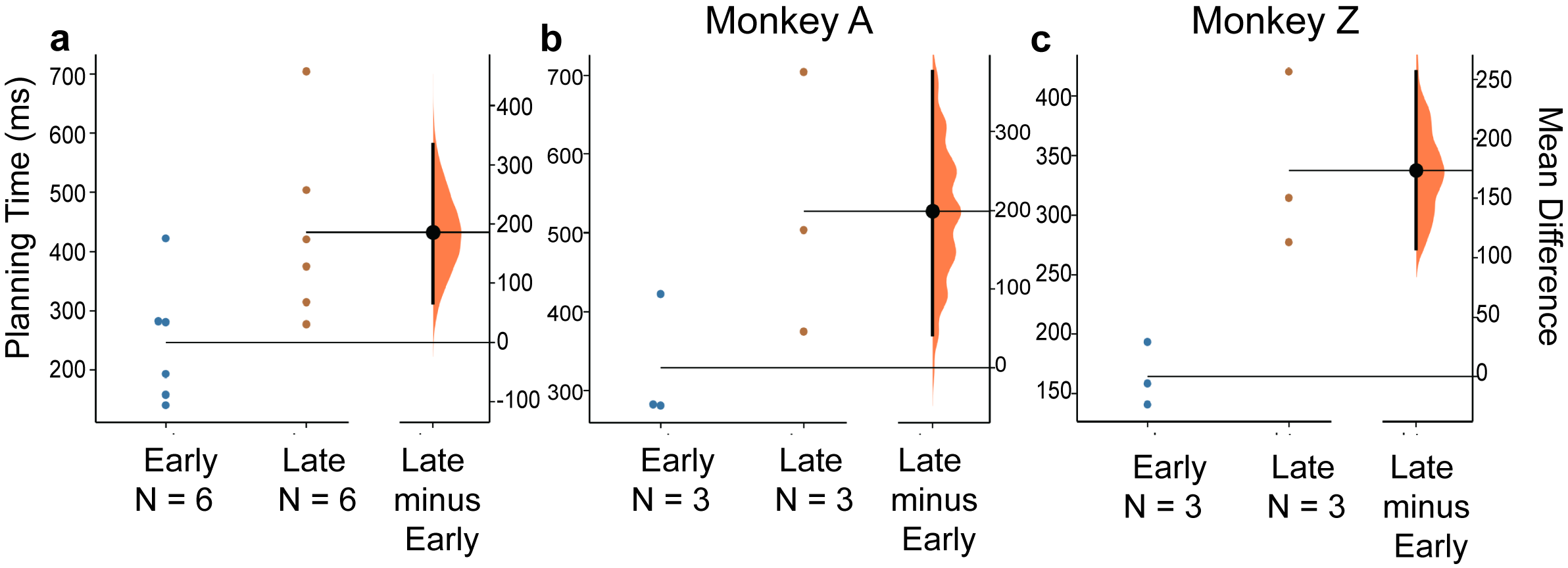

Supplement: Extended Data Figure 6-2 — The planning time of early and late saline sessions in the AVM-NC task. The mean interval time between early and late sessions was 14.3 d. a, The mean planning time of both monkeys between early (blue) and late (orange) sessions. b, c, The performance of Monkey A and Monkey Z. Download Figure 6-2, TIF file. [file enu-eN-NWR-0015-23-s08.tif]

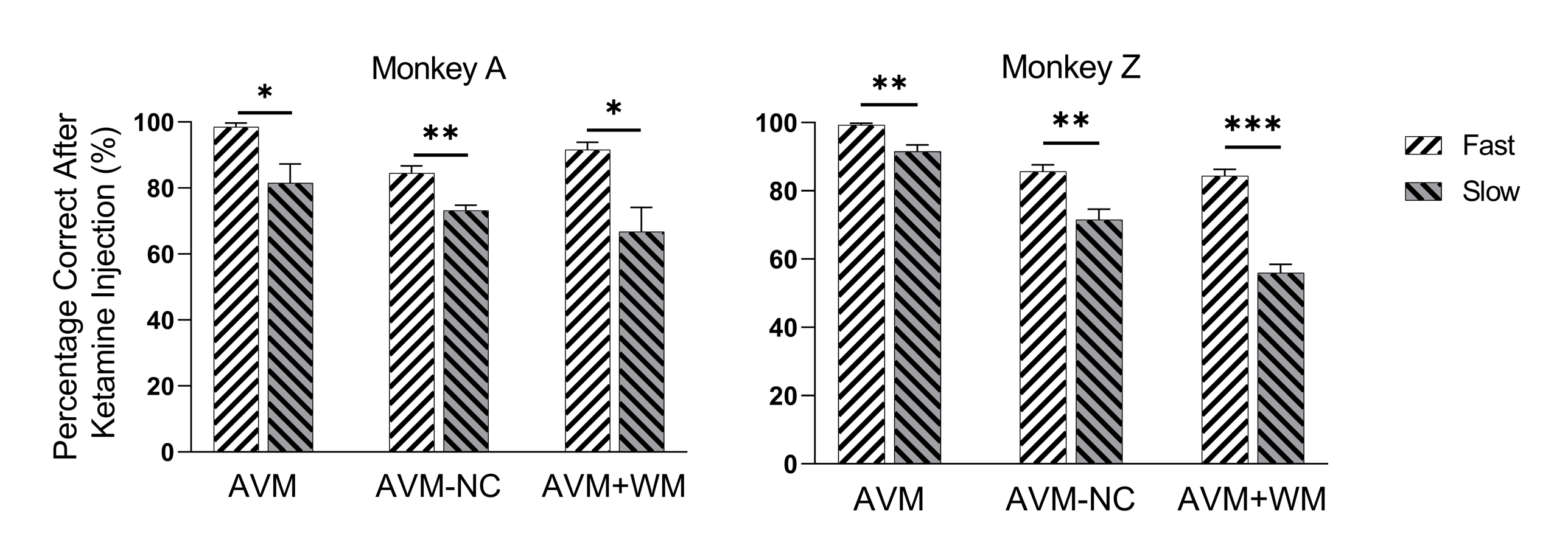

Supplement: Extended Data Figure 7-1 — The accuracy comparison of the fast response trials and slow response trials per subject. Trials were rearranged based on reaction time (planning time plus the movement time) following an injection of 0.8 mg/kg ketamine during a 60-min period. Specifically, trials were divided into two groups within each daily session: the fast response group (comprising the first quart of trials with the shortest reaction time) and the slow response group (comprising the last quart of trials with the longest reaction time). The figure shows a comparison of the accuracy between the fast response group (Fast) and the slow response group (Slow). All error bars correspond to the SEM. Significance levels are denoted as follows: *p < 0.05, **p < 0.01, and ***p < 0.001. Download Figure 7-1, TIF file. [file enu-eN-NWR-0015-23-s09.tif]

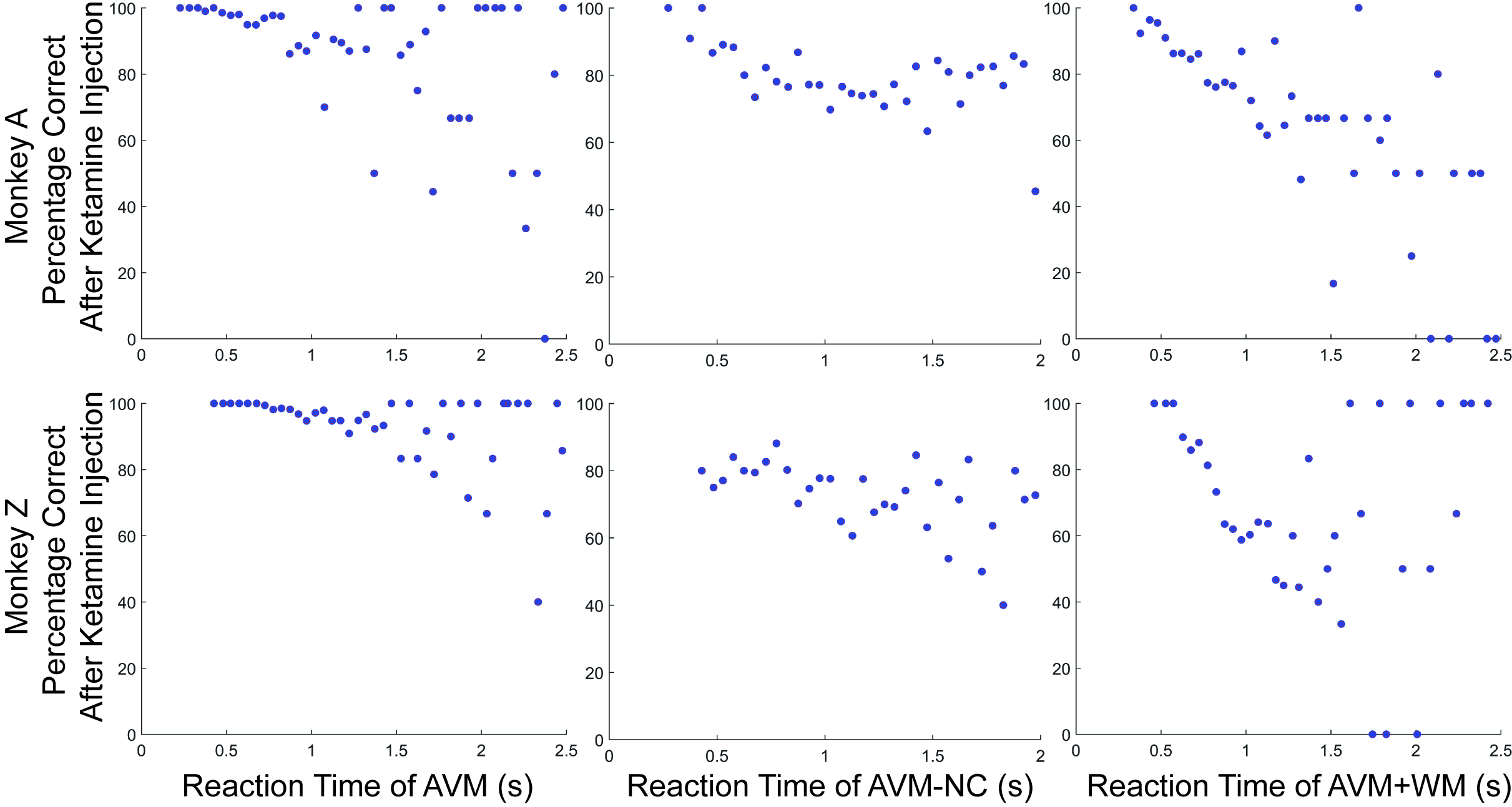

Supplement: Extended Data Figure 8-1 — The correlation between the accuracy and reaction time after administrating 0.8 mg/kg ketamine per subject. The blue dots in the figure represent the corresponding average accuracy within the reaction time. Download Figure 8-1, TIF file. [file enu-eN-NWR-0015-23-s10.tif]

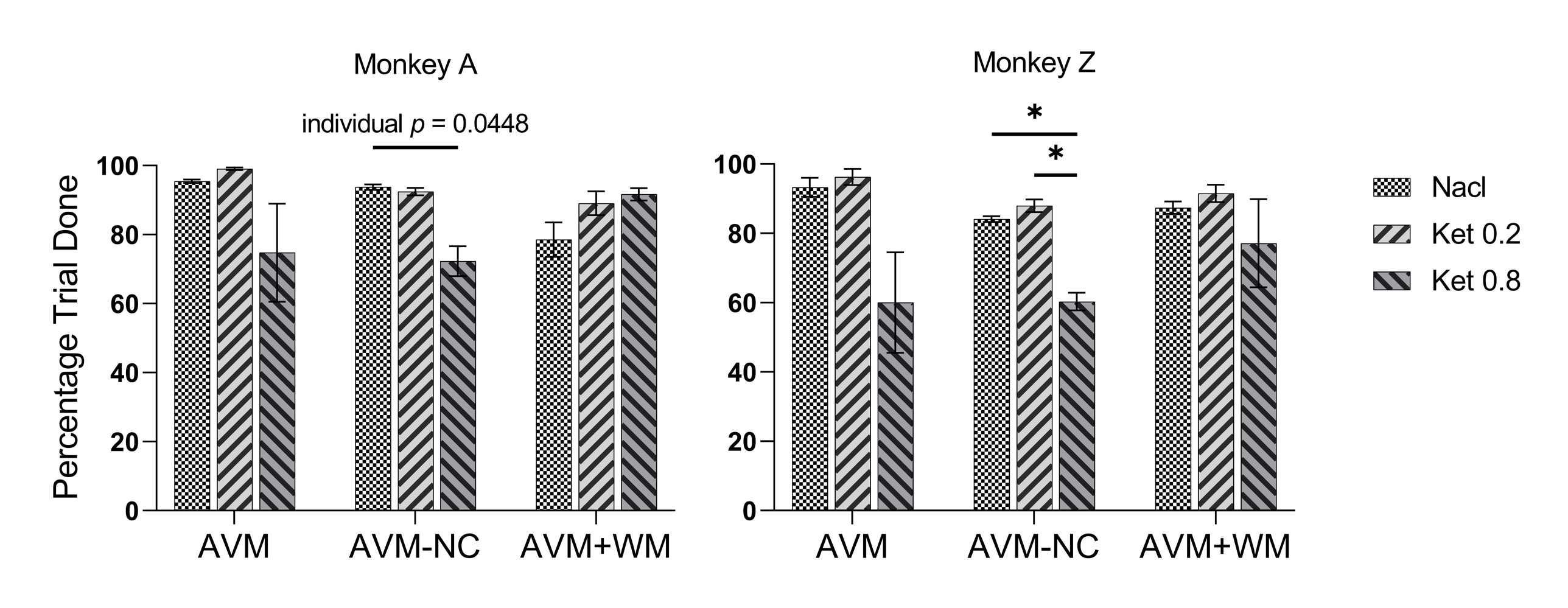

Supplement: Extended Data Figure 9-1 — The percentage of complete trials after injection per subject. The percentage trial done was defined as the ratio of completed trials to all trials (complete and incomplete trials). This figure shows the percentage of complete trials in early-postinjection phase under saline (Nacl), 0.2 mg/kg ketamine (Ket 0.2), and 0.8 mg/kg ketamine (Ket 0.8) in each task. The error bars indicate the SEM. Statistical significance is denoted by *p < 0.05. Download Figure 9-1, TIF file. [file enu-eN-NWR-0015-23-s11.tif]
