# Supplementary material for: Low-Dose Ketamine-Induced Deficits in Arbitrary Visuomotor Mapping in Monkeys
Source: eNeuro. 2023 Jun 23;10(6):ENEURO.0015-23.2023. doi: 10.1523/ENEURO.0015-23.2023 (PMC10309660; doi:10.1523/ENEURO.0015-23.2023)
Supplement: Extended Data Table 4-1 — Statistics for the Figure 4 in main text. Download Table 4-1, PDF file. [file enu-eN-NWR-0015-23-s12.pdf]

Table 4-1. Statistics for the Figure 4 in main text of paper

Figure	Sample Size(numbers of sessions in different conditions of three tasks)	Statistical Test Values	
**Fig 4a** Decision-make time in the AVM task	Saline<br>N=12<br><br>0.2 mg/kg ketamine<br>N=6<br><br>0.8 mg/kg ketamine<br>N=11	Ordinary one-way ANOVA, F (2, 26) = 8.575, p=0.0014	
		Tukey’s multiple comparisonstest	Adjusted P Value
		Nacl vs. Ket 0.2	0.9932
		Nacl vs. Ket 0.8	0.0025
		Ket 0.2 vs. Ket 0.8	0.0099
**Fig 4b** Movement time in the AVM task		Kruskal-Wallis, p=0.0045	
		Dunn's multiple comparisons test	Adjusted P Value
		Nacl vs. Ket 0.2	>0.9999
		Nacl vs. Ket 0.8	0.0104
		Ket 0.2 vs. Ket 0.8	0.0267
**Fig 4c** Decision-make time in the AVM-NC task	Saline<br>N=26<br><br>0.2 mg/kg ketamine<br>N=13<br><br>0.8 mg/kg ketamine<br>N=12	Ordinary one-way ANOVA, F (2, 48) = 43.61	P<0.0001
		Tukey’s multiple comparisons test	Adjusted P Value
		Nacl vs. Ket 0.2	0.8593
		Nacl vs. Ket 0.8	<0.0001
		Ket 0.2 vs. Ket 0.8	<0.0001
**Fig 4d** Movement time in the AVM-NC task		Ordinary one-way ANOVA, F (2, 48) = 5.717	P=0.0059
		Tukey's multiple comparisons test	Adjusted P Value
		Nacl vs. Ket 0.2	0.8539
		Nacl vs. Ket 0.8	0.0122
		Ket 0.2 vs. Ket 0.8	0.0100
**Fig 4e** Decision-make time in the AVM+WM task	Saline<br>N=23<br><br>0.2 mg/kg ketamine<br>N=8<br><br>0.8 mg/kg ketamine<br>N=8	Ordinary one-way ANOVA, F (2, 36) = 20.94	P<0.0001
		Tukey’s multiple comparisons test	Adjusted P Value
		Nacl vs. Ket 0.2	0.2792
		Nacl vs. Ket 0.8	<0.0001
		Ket 0.2 vs. Ket 0.8	0.0008
**Fig 4f** Movement time in the AVM+WM task		Ordinary one-way ANOVA, F (2, 36) = 3.252	P=0.0503
		Tukey's multiple comparisons test	Adjusted P Value
		Nacl vs. Ket 0.2	0.9920
		Nacl vs. Ket 0.8	0.0516
		Ket 0.2 vs. Ket 0.8	0.1053
